# OTUD1 inhibits osteoclast differentiation and osteoclastic bone loss through deubiquitinating and stabilizing PRDX1

**DOI:** 10.7150/thno.111360

**Published:** 2025-06-09

**Authors:** Xiaoyu Sun, Tong Wu, Shuhong Chen, Zheyu Zhao, Ruiwei Jia, Jun Ma, Lei Yin, Xingbei Pan, Yifan Ping, Yixin Mao, Lulu Ma, Yilin Ma, Wu Luo, Shengbin Huang, Guang Liang

**Affiliations:** 1 Institute of Stomatology, School and Hospital of Stomatology, Wenzhou Medical University, Wenzhou, China.; 2 Department of Periodontics, School and Hospital of Stomatology, Wenzhou Medical University, Wenzhou, China.; 3 Chemical Biology Research Center, School of Pharmaceutical Sciences, Wenzhou Medical University, Wenzhou, China.; 4 Department of Prosthodontics, School and Hospital of Stomatology, Wenzhou Medical University, Wenzhou, China.; 5 School of Pharmacy, Hangzhou Medical College, Hangzhou, Zhejiang 310012, China.

**Keywords:** Osteoclast differentiation, OTUD1, Deubiquitination, PRDX1, Mitochondrial dysfunction

## Abstract

**Rationale:** Bone homeostasis relies on a delicate equilibrium between bone formation by osteoblasts and bone resorption by osteoclasts. Disruption of this balance leads to various disorders, most notably osteoporosis. Deubiquitinating enzymes (DUBs), which cleave ubiquitin moieties from substrate proteins, play critical regulatory roles in bone pathophysiology. In this study, we explored the function of a DUB, ovarian tumor deubiquitinase 1 (OTUD1), in bone remodeling.

**Methods:** We examined the femur bone of *Otud1^+/+^* and *Otud1^-/-^* male mice using micro-CT analyses and histomorphometry. The potential functions and mechanisms of OTUD1 were explored in bone marrow-derived macrophages, RAW264.7 cells, and bone marrow stromal cells using RT-qPCR, western blotting and immunofluorescence. Additionally, we employed liquid chromatography-tandem mass spectrometry (LC-MS/MS) coupled with co-immunoprecipitation (Co-IP) to identify OTUD1-interacting proteins and substrates.

**Results:** Our results demonstrated a significant downregulation of both the gene and protein level of OTUD1 during osteoclastogenesis. Furthermore, both whole-body knockout and myeloid-specific deficiency of OTUD1 resulted in reduced bone mass in male mice, driven by enhanced osteoclast differentiation. Mechanistically, OTUD1 maintained the stability of peroxiredoxin 1 (PRDX1) by reversing K48-linked ubiquitination, thereby mitigating mitochondrial dysfunction and suppressing osteoclast differentiation. Consistent with these results, mitochondria-targeted ubiquinone (MitoQ), a mitochondria-targeted antioxidant, effectively suppressed bone mass loss in OTUD1-deficient male mice.

**Conclusions:** Our findings provided the first evidence that OTUD1 suppressed osteoclastogenesis by deubiquitinating PRDX1 and maintaining its stability, thereby offering a promising therapeutic approach for osteoclast-dependent bone diseases.

## Introduction

Bone homeostasis relies heavily on the continuous cycle of bone formation by osteoblasts and bone resorption by osteoclasts 1 Disrupted bone homeostasis, characterized by reduced osteoblastic bone formation and enhanced osteoclastic resorption, drives progressive bone loss. The resulting fragility fractures and chronic disability from bone diseases, such as osteoporosis, severely impair quality of life and impose substantial socioeconomic burdens, highlighting the critical importance of maintaining bone metabolic equilibrium 2. Targeted regulation of osteoclasts and osteoblasts has been proven effective in alleviating bone diseases. Therefore, elucidating the underlying molecular mechanisms is crucial for both understanding the pathogenesis of bone diseases and developing novel therapies 3, 4.

Bone metabolism is intricately governed by protein modulation, and stable regulation of protein fate is a prerequisite for efficient bone tissue repair 5. Ubiquitination and deubiquitination, as the post-translational modifications of proteins, are important for various pathophysiological activities. The ubiquitin-proteasome system (UPS) is responsible for degrading the majority of intracellular proteins, maintaining a delicate equilibrium between ubiquitination catalyzed by E3 ubiquitin ligases and deubiquitination facilitated by deubiquitinating enzymes (DUBs) 6, 7. The human genome encodes approximately 100 DUBs, which play pivotal roles in stabilizing targeted proteins by removing ubiquitin molecules 8, 9. DUBs are further categorized into seven subclasses: ubiquitin-specific proteases (USPs), ubiquitin C-terminal hydrolases, ovarian tumor proteases (OTUs), Machado-Josephin domain proteases, a motif interacting with the ubiquitin-containing DUB family, zinc finger proteins with a UFM1-specific peptidase domain/zinc finger containing Ub peptidase 1, and JAB1/MPN/Mov34 metalloenzyme 10, 11. Several USP subfamily members, including USP26 12, USP7 9, and USP34 13, have been reported to regulate bone homeostasis through distinct mechanisms. For example, USP26 promoted osteogenic differentiation of bone marrow mesenchymal stem cells while inhibiting osteoclastogenesis 12. USP7 exacerbated osteoporosis by deubiquitinating HMGB1 to stimulate osteoclast differentiation in peripheral blood monocytes 9. USP34 suppressed osteoclastogenesis through stabilization of IκBα 13. Notably, although the OTU deubiquitinase family has been implicated in inflammatory and immune diseases 14, 15. Only one member of the OTU family, OTUB1, was proved to promote osteogenesis via FGFR2 stabilization 16. The involvement of more OTU enzymes in bone metabolism remains largely unknown. Elucidating the mechanisms by which OTU enzymes mediate bone remodeling is essential for developing new deubiquitinase-targeted therapies for osteoporosis.

Here, we identified ovarian tumor deubiquitinase 1 (OTUD1), a member of the OTU subfamily, as a critical regulator of bone metabolism. Although previous studies implicated OTUD1 in inflammatory diseases such as cerebral ischemia and colonic inflammation 14, 17. Our findings revealed the novel role of OTUD1 in bone homeostasis. Specifically, whole-body knockout of OTUD1 and myeloid lineage-specific OTUD1 deficiency enhanced osteoclastogenesis and bone resorption in mouse femurs without affecting osteoblast-mediated bone formation. We further identified peroxiredoxin 1 (PRDX1) as a key substrate of OTUD1. As an essential antioxidant enzyme, PRDX1 scavenges reactive oxygen species (ROS) and alleviates mitochondrial dysfunction 18. Mechanistically, OTUD1 removed K48-linked ubiquitination from PRDX1, thereby stabilizing PRDX1, reducing oxidative stress and mitochondrial damage, and ultimately suppressing osteoclast differentiation. This study is the first to elucidate the role of OTUD1 in osteoclast-mediated bone loss, thereby providing a promising therapeutic strategy for treating osteoclast-related bone diseases.

## Material and Methods

### Human femoral tissue samples

With informed patient consent, femoral tissue samples from both healthy individuals and patients with osteoporosis undergoing knee arthroplasty were obtained from the Bone Disease Center of the Second Affiliated Hospital of Wenzhou Medical University. This experiment was approved by the Ethics Committee in Clinical Research (ECCR) of the Second Affiliated Hospital of Wenzhou Medical University to use clinical biopsy specimens (Approval No.2025IIT No. 26), and informed consent was obtained from the patients. All the studies followed the Declaration of Helsinki of 1975, amended in 2008 19. The patient information was shown in Table S1.

### Mice and animal experiments

All the experiments involving animals were approved by the Animal Ethics Committee of Wenzhou Medical University (Approval No. WYDW2019-0665) and conducted in accordance with the Guide for the Care and Use of Laboratory Animals (National Institutes of Health, USA). Age- and sex-matched whole-body knockout male mice (*Otud1^-/-^*) and the *Otud1^+/+^* littermates were constructed, and the treatment groups were assigned randomly. The animals were kept under the following housing conditions: 12-h dark and 12-h light cycle, 20 - 24 °C and 30% - 70% humidity.

Bone marrow transplantation: Mice with chimeric bone marrow were developed. The recipient mice were irradiated with a dose of 6 Gy. Subsequently, 5.0 × 10⁶ bone marrow cells isolated from the femur and tibia of *Otud1*^-/-^or *Otud1^+/+^* mice were injected into the donors via tail vein injection.

Mitochondria-targeted ubiquinone (MitoQ) treatment: A total of 20 *Otud1^+/+^* mice that received *Otud1*^-/-^ bone marrow (*Otud1*^-/-^→*Otud1^+/+^*) were randomly divided into the control group (administered phosphate buffered solution 100 μL) and MitoQ treatment group (administered 5 mg/kg, once daily for 2 weeks (Medchemexpress, NJ, USA)). Following the treatment period, the mice were euthanized, and blood and bone tissues were collected for further analysis.

Lipopolysaccharide (LPS)-induced cranial bone loss model: LPS-induced calvarial osteolysis model was established in *Otud1*^+/+^ and *Otud1*^-/-^ mice. Specifically, 8-week-old male mice were administered subcutaneous injections of 100 μL 5 mg/kg LPS or PBS per day. These injections were administered around the sagittal suture of the skull. The mice were sacrificed on day 9, and calvariae were collected for further analysis.

### Micro-computed tomography (Micro-CT) test

Femur tissues and calvariae were fixed in 4% paraformaldehyde (PFA). Micro-CT analysis was performed using a Skyscan 1176 instrument (Bruker, Kontich, Belgium) at 50 kVp, 450 μA and a resolution of 9 μm. The obtained images were reconstructed using NRecon software (v.1.7.1.0, Bruker). The region of interest (ROI) for the trabecular bone was defined as the area located 0.5 mm from the growth plates, and 200 slices (2 mm) were reconstructed. Trabecular bone parameters, including bone volume per tissue volume (BV/TV), trabecular thickness (Tb. Th), trabecular number (Tb. N), and trabecular separation (Tb. Sp), were analyzed using CTAn (v.1.16, Bruker).

### Histological analysis

Bone samples were dissected and fixed in 4% PFA for 48 h, and decalcified for 7 days. The specimens were then cut into 5-μm-thick sections. Following deparaffinization and rehydration, bone sections were subjected to hematoxylin and eosin (H&E) staining (G1120, Solarbio, China) and tartrate-resistant acid phosphatase (TRAP) staining (G1492, Solarbio, China). Images were captured using a Nikon Eclipse Ti-U microscope (Nikon, Japan) and analyzed with NIS-Elements software (v.4.5000.1117.0). TRAP-positive multinucleated cells containing three or more nuclei were counted as osteoclasts using a light microscope.

### Immunohistochemistry

Dewaxed and rehydrated sections were subjected to antigen retrieval in a boiling water bath using 0.01M citrate buffer (pH 6.0) for 2 min. Endogenous peroxidases were blocked with 0.3% hydrogen peroxidase for 10 min, followed by incubation with 5% normal goat serum for 30 min to prevent nonspecific antigen binding. The sections were incubated with primary antibodies against OTUD1 (orb185712, 1:600, Biorbyt), RUNX2 (MA541185, 1:300, Thermo Fisher Scientific), 8-OHDG (ab48508, 1:600, Abcam) and PRDX1 (ab109498, 1:600, Abcam) for 2 h at room temperature. HRP457 -conjugated secondary antibodies (Horseradish Peroxidase, Cat# 457, ZSGB-BIO, China) and 3,3'-Diaminobenzidine were used for color development. The sections were then dehydrated across an alcohol gradient and sealed with neutral gum. All images were captured under bright-field illumination using an epifluorescence microscope equipped with a digital camera (Nikon, Tokyo, Japan). Image J software (National Institutes of Health, Bethesda, MD, USA) was used to quantify positively stained areas of RUNX2 and PRDX1.

### Immunofluorescence microscopy

The decalcified samples were dehydrated in serial sucrose solutions and embedded in optimal cutting temperature (OCT) compound (Tissue-Tek, Sakura, Japan). OCT-embedded samples were cryosectioned at 8 μm using a cryostat (Leica CM1850) followed by staining. Frozen sections were used for immunofluorescence staining. The slides were fixed in cold methanol and permeabilized with 0.5% Triton-X. The slides were blocked with 5% bovine serum albumin for 30 min and incubated overnight with primary antibodies. The primary antibodies used were anti-OTUD1 (orb185712, 1:600, Biorbyt), anti-CTSK (ab37259, 1:100, Abcam), and anti-PRDX1 (ab109498, 1:200, Abcam). Fluorophore-conjugated secondary antibodies were used for the final detection. The slides were counterstained with 4',6-Diamidino-2-Phenylindole (DAPI) for 5 min. Images were captured using a fluorescence microscope (Nikon, Japan).

### Enzyme-linked immunosorbent assay

Blood samples from mice were used to determine the protein levels of C-terminal telopeptide of collagen type 1 (CTX-I) (Cat#: MU30091, Bioswamp, Wuhan, China) and procollagen I amino-terminal propeptide (PINP) (Cat#: MU30602, Bioswamp, Wuhan, China) according to the manufacturer's protocol.

### Cell culture

Bone marrow-derived macrophages (BMDMs) were isolated from the femurs and tibias of *Otud1^+/+^* and *Otud1*^-/-^ mice. Bone marrow was harvested by flushing the femurs and tibias of the mice with α-MEM (Minimum Essential Medium, Gibco) using a 25-gauge needle. The collected bone marrow was then treated with red cell lysis buffer (NH4CL2009, TBD Science, China) for 3 min, followed by centrifugation, resuspension, and filtration through a 40 µm cell strainer. Cells were plated at a density of 1 × 10⁶ cells/mL in medium supplemented with 10% fetal bovine serum (FBS), 1% penicillin/streptomycin, and macrophage colony-stimulating factor (M-CSF) (25 ng/mL, #ZD0000000G, Bio-Rad). Cultures were maintained at 37°C under 5% CO₂ for 7 days to induce macrophage differentiation.

Bone marrow stromal cells (BMSCs) were isolated from the femur and tibia of *Otud1^+/+^* and *Otud1*^-/-^ male mice. Briefly, we isolated the femur and tibia of mice and flushed the bone marrow cavity using a syringe. The cells were lysed in red blood cell lysate buffer (NH4CL2009, TBD Science, Tianjin, China) and cultured in α-MEM (C12571500BT, Gibco, USA) containing 10% FBS and 1% penicillin / streptomycin solution at 37°C under 5% CO_2_. The medium was replaced every two days. BMSCs at passages 3 to 5 were used in the subsequent experiments.

RAW264.7 cells (TIB-71, ATCC, USA) and HEK-293T cells (CRL-1573, ATCC, USA) were obtained from American Type Culture Collection. RAW264.7 and HEK-293T cells were cultured in α-MEM (C12571500BT, Gibco, USA) containing 10% FBS, 100 U/mL streptomycin, and 100 U /mL penicillin.

### Gene knockdown and overexpression

Gene silencing and overexpression in cells were achieved by transfecting specific small interfering RNA (siRNA) and plasmids. The target sequence of the siRNA was as follows: 5′-CAGAUGCUGAAUGUGAAUAUACTT-3′ for *Otud1*. Plasmids encoding FLAG-OTUD1, FLAG-OTUD1(C320S), GFP-PRDX1, GFP-PRDX1△1-40, GFP-PRDX1△41-156, GFP-PRDX1△157-199, GFP-PRDX1(K7R), GFP-PRDX1(K16R), GFP-PRDX1(K120R), GFP-PRDX1(K136R), HA-Ub, HA-K48, and HA-K63 were constructed by Genechem. Transfection of RAW264.7 and HEK-293T cells with siRNA and plasmids was performed using Lipofectamine^TM^ 3000 (Thermo Fisher Scientific, USA).

### Cell treatment

BMDMs were defined as adherent cells at the bottom of the culture dish. For osteoclast differentiation, BMDMs were stimulated with M-CSF (25 ng/mL, #ZD0000000G, Bio-Rad) and receptor activator of nuclear factor kappa-B ligand (RANKL) (50 ng/mL, CWA2724072, Bio-Techne) for 4 days. RAW 264.7 cells were treated with RANKL (50 ng/mL, CWA2724072, Bio-Techne) for 4 days to induce osteoclastogenesis. During differentiation, BMDMs were exposed to mitoQ (HY-100116A, MCE, 0.2 μM) for 4 days. MitoQ was dissolved in dimethyl sulfoxide (DMSO) to a final concentration of 0.1% (v/v).

### Proteomics analysis

Protein extraction and trypsin digestion. Cell samples were lysed in cold lysis buffer (8 M urea and 1% protease inhibitor cocktail). The supernatant was collected, and the protein concentration was determined. The protein solution (200 μg for each sample) was reduced with dithiothreitol (5 mM, 56°C, 30 min) and alkylated with iodoacetamide (11 mM, room temperature, 15 min) in darkness. The protein solution was first digested with trypsin (1:50 trypsin to protein mass ratio, 37°C, overnight) and then digested with trypsin again (1:100 trypsin to protein mass ratio, 37°C, 4 h).

Ubiquitinated peptide enrichment and liquid chromatography-tandem mass spectrometry (LC-MS/MS). The peptides obtained by digestion were used for ubiquitin peptide enrichment (95%) and protein quantification (5%). The peptides used for ubiquitin peptide enrichment were dissolved in NaCl-EDTA-Tris-Nonidet P-40 buffer and incubated with anti-diglycine remnant (K-ε-GG) pan antibody beads (CST and PTM Biolabs; 4°C, overnight) with gentle shaking. LC-MS/MS and subsequent combined ubiquitinome, proteome and interactome analyses were performed by PTM Biolabs. Based on mass spectrometry data, we identified potential OTUD1 substrate proteins by evaluating their peptide coverage rate, and number of unique peptide, and FLAG/IgG ratios.

### Western blotting and co-immunoprecipitation (Co-IP)

Total protein was extracted from the cells using a lysis buffer (Boster Biological Technology, Pleasanton, CA, USA, AR0103). Proteins were separated using 10% sodium dodecyl sulfate polyacrylamide gel electrophoresis (SDS-PAGE) and transferred to polyvinylidene fluoride membranes. Before adding the specific primary antibodies, the membranes were blocked with 5% skim milk for 1.5 h at room temperature. Then, the membranes were incubated overnight at 4°C with primary antibodies as follows: anti-OTUD1 (orb185712, 1:1000, Biorbyt), anti-PRDX1 (ab109498, 1:200, Abcam), anti-HA (Cat. #3724, 1:1,000, CST), anti-GFP (Cat#2956, 1:1000, CST), anti-FLAG (Cat#14793, 1:1,000, CST), anti-NFATC1(sc-7294, 1:1,000, Santa), anti-CTSK (ab37259, 1:1000, Abcam), and anti-GAPDH (Cat#2118, 1:1,000, CST). Protein bands were detected by incubating the membrane with horseradish peroxidase-conjugated secondary antibodies and enhanced chemiluminescence reagent (Bio-Rad, Hercules, CA, USA). Band densities were quantified using the Image J software and normalized to the loading controls.

For Co-IP assays, treated cell lysates were incubated with target antibodies overnight at 4°C. Then the proteins were immunoprecipitated with Protein A + G Agarose (P2012, Beyotime, Shanghai, China) at 4°C for 4 h. The samples were immunoblotted to detect the co-precipitated proteins. Total lysates were subjected to western blot analysis as input controls. Protein interactions were quantified using Image J software.

### TRAP staining

The cells were washed with PBS and fixed with 4% paraformaldehyde for 15 min. After washing with PBS, the cells were incubated in a reaction mixture of the leukocyte acid phosphatase kit (Cat#: 387A-1KT, Sigma, German) at 37˚C for 3 h, away from light. The cells were washed thrice with distilled water and TRAP-positive multinucleated cells containing five or more nuclei were visualized using light microscopy and counted as mature osteoclasts.

### Alkaline phosphatase (ALP) activity analysis

BMSCs were cultured in 24-well plates and stimulated for 7 days in an osteogenic differentiation medium (MUXMX-90021, OriCell). The cells were fixed with PFA, washed, and mixed with 5-Bromo-4-Chloro-3-Indolyl Phosphate/Nitro Blue Tetrazolium (BCIP/NBT, Beyotime, China). The reaction was terminated by washing the cells with distilled water. Images were captured using a light microscope.

### Real-time quantitative polymerase chain reaction (RT-qPCR)

Total RNA was isolated and purified from the cells using TRIzol reagent. Purified RNA was reverse transcribed using the PrimeScript RT reagent kit (Cat#: 11201ES03, Yeasen, Shanghai, China) to generate cDNA. RT-qPCR was performed using a SYBR Green reagent kit (Cat#: R223-00, Vazyme, Nanjing, China). An RT2 Profiler Custom RT-qPCR array was used to examine the mRNA levels of genes associated with the OTU deubiquitinase family according to the manufacturer's protocol (Bio TNT, QMPGG2132, Shanghai, China). The primers used for RT-qPCR analysis are listed in Table S2.

### Prediction of ubiquitination sites via GPS-Uber

We employed a tool named GPS-Uber^23^ to predict ubiquitination sites (Table S3), which is available at http://gpsuber.biocuckoo.cn/.

### Mitochondrial imaging assays

BMDM and RAW264.7 cells were cultured in 96-well plates and stimulated under different conditions. The cells were then incubated with tetramethylrhodamine methyl Ester (TMRM) probes (1 μM, I34361, Thermo Fisher Scientific, USA) and Mitogreen probes (1 μM, M7514, Thermo Fisher Scientific, USA) to detect mitochondrial membrane potential (MMP) and Mitosox probes (1 μM, M36008, Thermo Fisher Scientific, USA) and Mitogreen probesfor detection of mitochondrial reactive oxygen species (mtROS) levels. Images were captured using a fluorescence microscope (Zeiss) and quantified using NIH ImageJ software.

### Determination of intracellular ROS

BMDM and RAW264.7 cells were cultured in 96-well plates and stimulated under different conditions. Cells were then incubated with 2',7'-Dichlorodihydrofluorescein Diacetate (DCFH-DA, S0033S, 1:1,000, Beyotime, China) for 30 min at 37°C and washed three times with PBS. Finally, the cells were observed under a fluorescence microscope (Zeiss) and quantified using NIH ImageJ software.

### Adenosine triphosphate (ATP) evaluation

BMDM and RAW264.7 cells were cultured in 96-well plates and stimulated under different conditions. Whole-cell lysates were prepared using lysis buffer from an ATP assay kit (Beyotime, China). After centrifugation, the supernatants were collected. Luminescence was measured using a luminometer and ATP detection buffer.

### Immunofluorescence staining

RAW264.7 cells cultivated on slides were transfected with plasmids expressing FLAG-tagged OTUD1 or GFP-tagged PRDX1. The cells were fixed with 4% PFA for 10 min and permeabilized with 0.1% Triton X-100 for 5 min at room temperature. Next, the cells were blocked with 5%bovine serum albumin for 30 min at room temperature and incubated with anti-OTUD1 (orb185712, 1:600, Biorbyt, UK) and anti-PRDX1 (ab109498, 1:200, Abcam, USA) antibodies overnight at 4°C, followed by the corresponding secondary antibody for 1 h at 37°C. The slides were mounted with DAPI-containing medium and coverslipped for fluorescence microscopy. Nikon Eclipse Ti-U Microscope (Nikon, Japan) with NlS-Elements software (y4.5000.1117.0) and Zeiss Axio Scope A1 Microscope (ZEISS, German) were used to acquire images.

### Statistical analysis

Data are presented as mean ± standard error of the mean (SEM). The number of replicates (*n*) is indicated in the figure legends. The significance of the differences was evaluated using a two-tailed t-test or two-way analysis of variance (ANOVA), followed by Tukey's multiple comparison test for comparisons among three or more groups. Statistical significance was set at *P* < 0.05. In the figures, asterisks denote statistical significance (^*^p < 0.05; ^**^p < 0.01; ^***^p < 0.001). Statistical analyses were performed using GraphPad Prism 9.

## Results

### OTUD1 deficiency contributed to bone loss in mice

**Figure 1:**
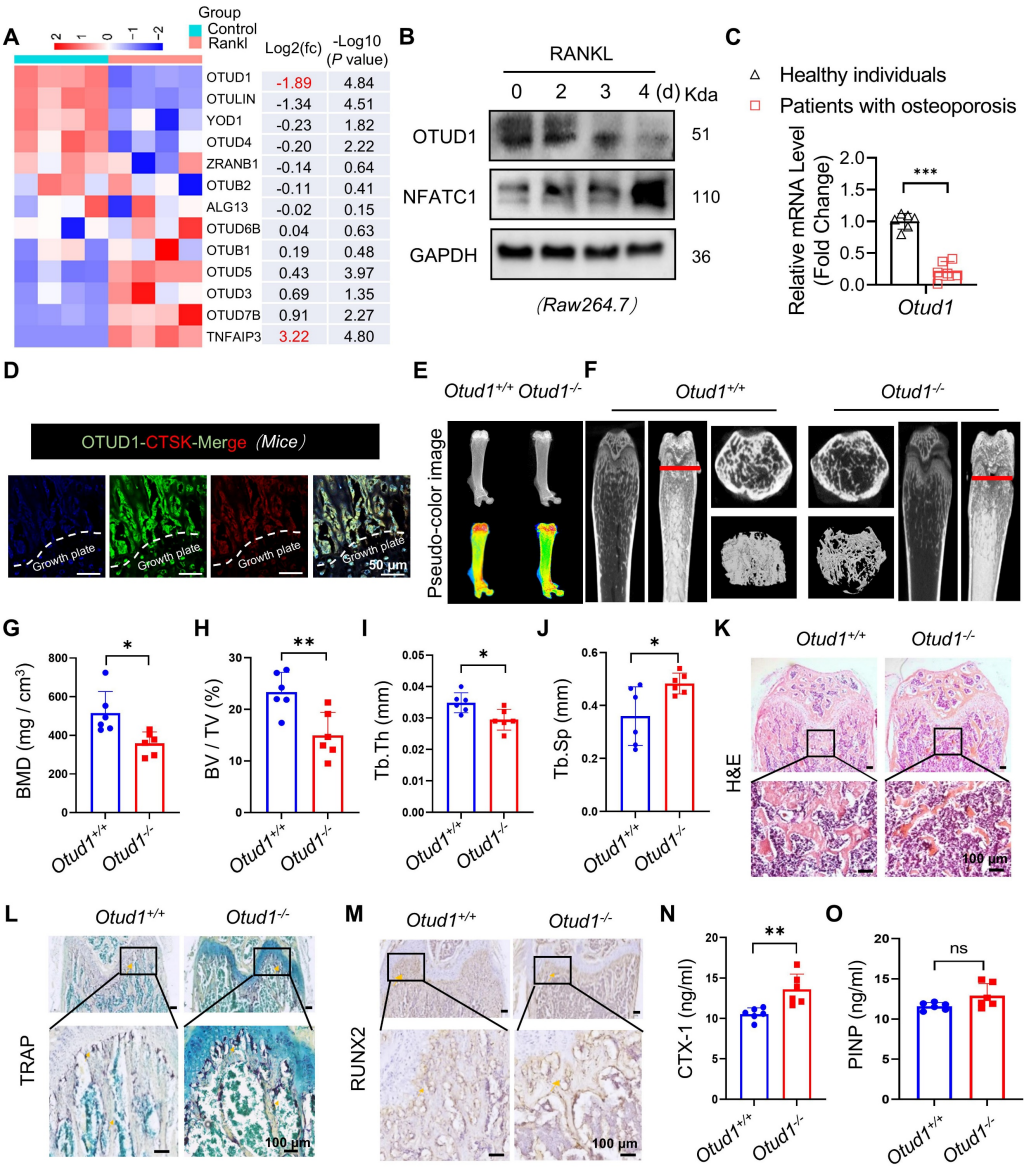
** OTUD1 deficiency reduced femur bone mass in mice. (A)** RT-qPCR array results revealed the mRNA levels of OTU family members in BMDMs challenged with or without RANKL (n = 4). (**B**) Representative immunoblot of OTUD1 in RAW264.7 after RANKL-induction (n = 3). (**C**) mRNA levels of *Otud1* in the femoral bone of healthy individuals and patients with osteoporosis. (**D**) Immunofluorescence (IF) images of OTUD1 (green) and CTSK (red) in the femurs of healthy mice (n = 3, 50 μm). (**E**) Representative pseudo-color image of X-ray of femur in *Otud1*^-/-^ and *Otud1^+/+^* mice, used to analyze the distribution of bone mineralization. Blue, yellow, and red indicate high-density areas, and green indicates low-density areas (n = 6). **(F-J)** Representative micro-CT images of the distal trabecular bone of femurs from 8-week-old *Otud1^+/+^* and *Otud1*^-/-^ mice **(F)**. (n = 6). Quantification analysis included BMD **(G)**, bone volume/tissue volume ratio (BV/TV) **(H)**, trabecular thickness (Tb. Th) **(I)**, and trabecular separation (Tb. Sp)** (J)** (n = 6). **(K)** H&E staining of femurs from* Otud1^+/+^* and *Otud1*^-/-^ mice, with the boxed area in the first panel magnified below (n = 6, 100 μm). **(L)** Representative image of TRAP staining of distal trabecular and cortical bone in femurs from *Otud1*^-/-^ and *Otud1^+/+^* mice (n = 6, 100 μm). **(M)** Immunohistochemical staining of RUNX2 in the femurs of mice (n = 5, 100 μm). **(N-O)** ELISA measurement of CTX-1 **(N)** and PINP **(O)** levels in the serum of *Otud1*^-/-^ and *Otud1^+/+^* mice (n = 6). Data are presented as mean ± SEM. *p < 0.05, **p < 0.01, ***p < 0.001, ns: no significant.

Upon RANKL stimulation, BMDMs undergo fusion to form osteoclasts, which are responsible for bone resorption 1. To investigate the involvement of OTU subfamily members in bone remodeling, we conducted RT-qPCR analysis of BMDMs challenged with or without RANKL. Notably, among all OTU subfamily members, OTUD1 exhibited the most pronounced downregulation, while *Tnfaip3* showed the strongest upregulation in response to RANKL stimulation. Studies have confirmed that TNFAIP3 ameliorated abnormal subchondral bone remodeling 20 and acted as a regulator of RANK-induced NF-κB signaling to control osteoclast differentiation 21. Therefore, OTUD1 piqued our interest in the present study (Figure 1A). We found that the protein levels of OTUD1 were progressively downregulated during osteoclastogenesis (Figure 1B). Notably, the gene expression of OTUD1 was significantly downregulated in the femoral bone tissue of patients with osteoporosis compared to healthy controls (Figure 1C and Table S1). Furthermore, immunofluorescence analysis of healthy mouse femurs demonstrated the co-localization of OTUD1 with cathepsin K (CTSK), a well-established marker of osteoclast 22 (Figure 1D). These results suggested a potential regulatory role of OTUD1 in bone metabolism.

We generated age- and sex-matched whole-body OTUD1 knockout male mice (*Otud1^-/-^*) and *Otud1^+/+^* littermates (Figure S1A). Whole-mount skeletal staining showed comparable skeletal sizes and limb skeletal elements, including the lengths of the humerus, femur, radius, and tibia, between *Otud1*^-/-^ and *Otud1^+/+^* postnatal Day 0 (P0) embryos (Figure S1B). Additionally, Alizarin red and Alcian blue double staining did not reveal any significant differences in bone formation in the skulls and limbs of *Otud1^-/-^* and *Otud1^+/+^* P0 embryos (Figure S1C). Similarly, for 8-week-old mice, no significant differences were observed in body length, weight, and femur and tibia lengths between *Otud1^-/-^* mice and their *Otud1^+/+^* littermates (Figure S1D-G). Collectively, these findings indicated that OTUD1 deficiency did not affect P0 embryonic development.

However, pseudo-color X-ray imaging revealed that, at 8 weeks of age, OTUD1 loss induced a notable reduction in femur bone mass (Figure 1E). Micro-CT analysis revealed that *Otud1^-/-^* mice presented decreased bone mineral density (BMD), BV/TV, and Tb. Th, and increased Tb. sp compared to that in the control mice (Figure 1F-J). H&E staining further highlighted osteopenic phenotypes in *Otud1^-/-^* mice, featuring thinner and less abundant trabecular bone (Figure 1K). TRAP staining demonstrated that OTUD1 loss increased the number and area of osteoclasts in the femur (Figure 1L and Figure S2A). Furthermore, OTUD1 deficiency led to elevated mRNA levels of nuclear factor of activated T Cells 1 (*Nfatc1*) in mouse femurs, a key factor required for osteoclast differentiation (Figure S2B). To determine whether OTUD1 affects osteoblast lineage, we investigated the number of RUNX2^+^ osteoblasts. The results indicated no significant difference between *Otud1^-/-^* mice and their *Otud1^+/+^* littermates in terms of the number of RUNX2^+^ osteoblasts (Figure 1M and Figure S2C) and the mRNA levels of the bone formation marker genes *Ocn* and* Alp* (Figure S2D-E). Furthermore, analysis revealed increased serum levels of bone resorption marker CTX-1 and comparable levels of bone formation biomarker N-terminal PINP in *Otud1^-/-^* mice compared to their *Otud1^+/+^* littermates (Figure 1N-O). These data indicated that the defective bone metabolism observed in *Otud1^-/-^* mice was primarily attributed to enhanced bone resorption, rather than delayed bone formation.

### Myeloid cell-specific OTUD1 deficiency contributed to bone loss in mice

**Figure 2:**
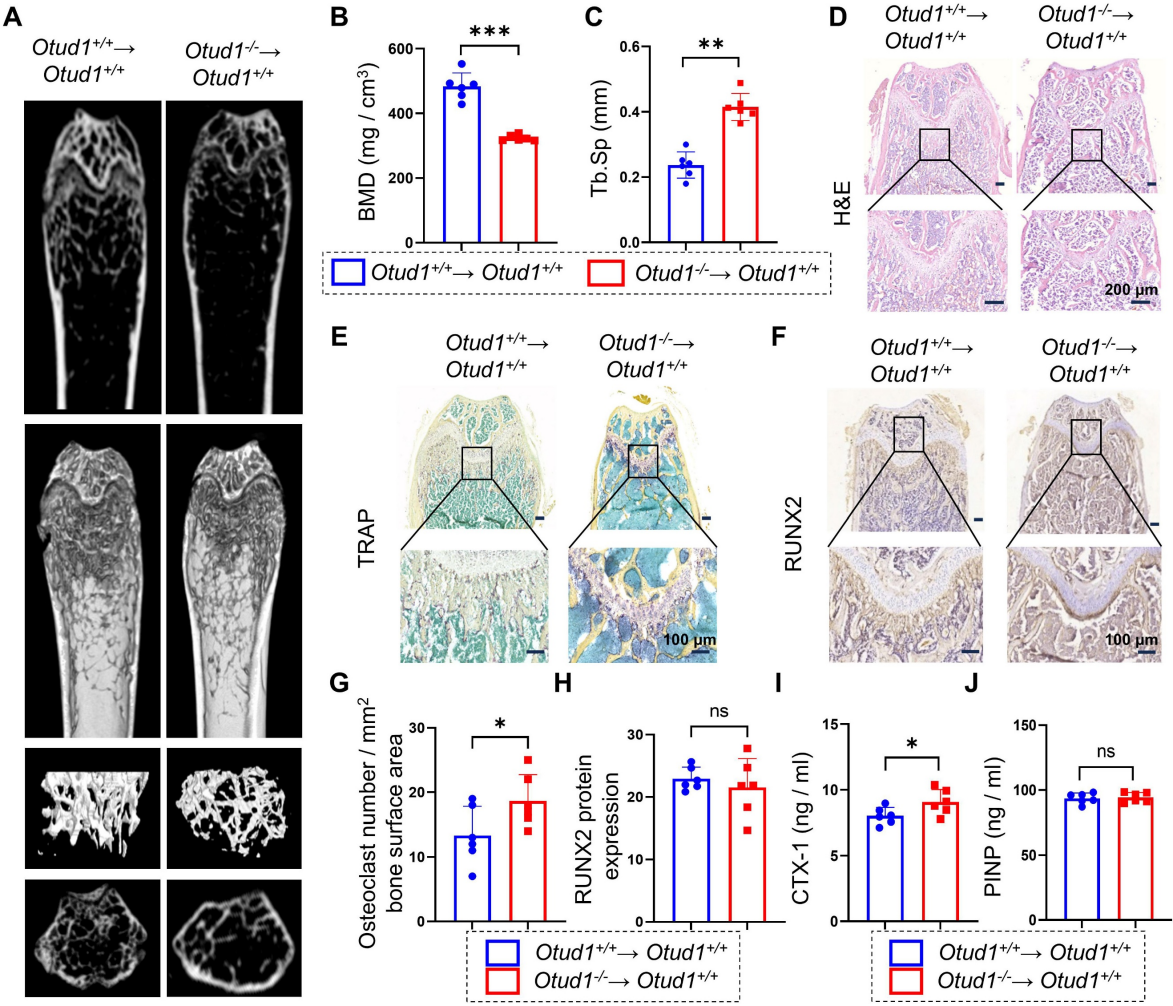
** OTUD1 from myeloid cells contributed to bone loss in mice. (A)** Representative micro-CT images of the distal trabecular bone in femurs from *Otud1^+/+^* mice receiving *Otud1^+/+^* or *Otud1*^-/-^ bone marrow (n = 6). **(B-C)** Quantitative analysis of BMD** (B)**, Trabecular separation (Tb. Sp) **(C)** (n = 6). **(D)** H&E staining of femurs from *Otud1^+/+^* mice receiving *Otud1^+/+^* or *Otud1*^-/-^ bone marrow, with the boxed area in the first panel magnified below (n = 6, 200 μm). **(E)** Representative image of TRAP staining of distal trabecular and cortical bone of femurs from *Otud1^+/+^* mice receiving *Otud1^+/+^* or *Otud1*^-/-^ bone marrow, with the boxed area in the first panel magnified below (n = 6, 100 μm). **(F)** Immunohistochemical staining for RUNX2 in femurs, with the boxed area in the first panel magnified below (n = 6, 100 μm). **(G)** Quantitative analysis of TRAP^+^ osteoclasts in femurs (n = 6). **(H)** Quantitative analysis of immunohistochemical staining of femurs from *Otud1^+/+^* mice receiving *Otud1^+/+^* or *Otud1*^-/-^ bone marrow (n = 6). **(I-J)** ELISA measurements of CTX-1 **(I)** and PINP **(J)** levels in the serum of *Otud1^+/+^* mice receiving *Otud1^+/+^* or *Otud1*^-/-^ bone marrow (n = 6). Data are presented as mean ± SEM. *p < 0.05, **p < 0.01, ***p < 0.001, ns: no significant.

Osteoclasts are derived from the hematopoietic lineage of cells derived from bone-marrow myeloid precursors. Bone loss driven by OTUD1 deficiency led us to hypothesize that OTUD1 in the bone marrow may contribute significantly to bone resorption. To test this hypothesis, we generated myeloid cell-specific OTUD1-deficient mice using bone marrow transplantation (Figure S3). Micro-CT analysis and histological staining demonstrated that, compared to *Otud1^+/+^* mice receiving *Otud1^+/+^* bone marrow (*Otud1^+/+^*→ *Otud1^+/+^* group), those receiving *Otud1*^-/-^ bone marrow (*Otud1*^-/-^→ *Otud1^+/+^* group) exhibited distinct osteogenic phenotypes, characterized by reduced BMD and increased Tb. Sp (Figure 2A-C), osteogenic phenotype (Figure 2D), and increased the number and area of TRAP-positive osteoclasts (Figure 2E, G). However, no significant difference was observed in the level of runt-related transcription factor-2 (RUNX2) in the femurs between the two groups (Figure 2F, H). Consistently, myeloid cell-specific deletion of OTUD1 resulted in elevated serum levels of CTX-1, whereas the level of PINP remained unaffected (Figure 2I-J). Additionally, we isolated BMSCs from *Otud1^-/-^* and *Otud1^+/+^* mice and observed no significant differences in ALP activity and the mRNA expression of osteoblast differentiation markers (*Alp*,* Ocn*, and *Osx*) between the two groups (Figure S4). Collectively, the specific deficiency of OTUD1 in myeloid cells, which are osteoclast precursors, aggravated bone resorption.

### OTUD1 suppressed osteoclastogenesis and osteoclast differentiation* in vitro*

**Figure 3:**
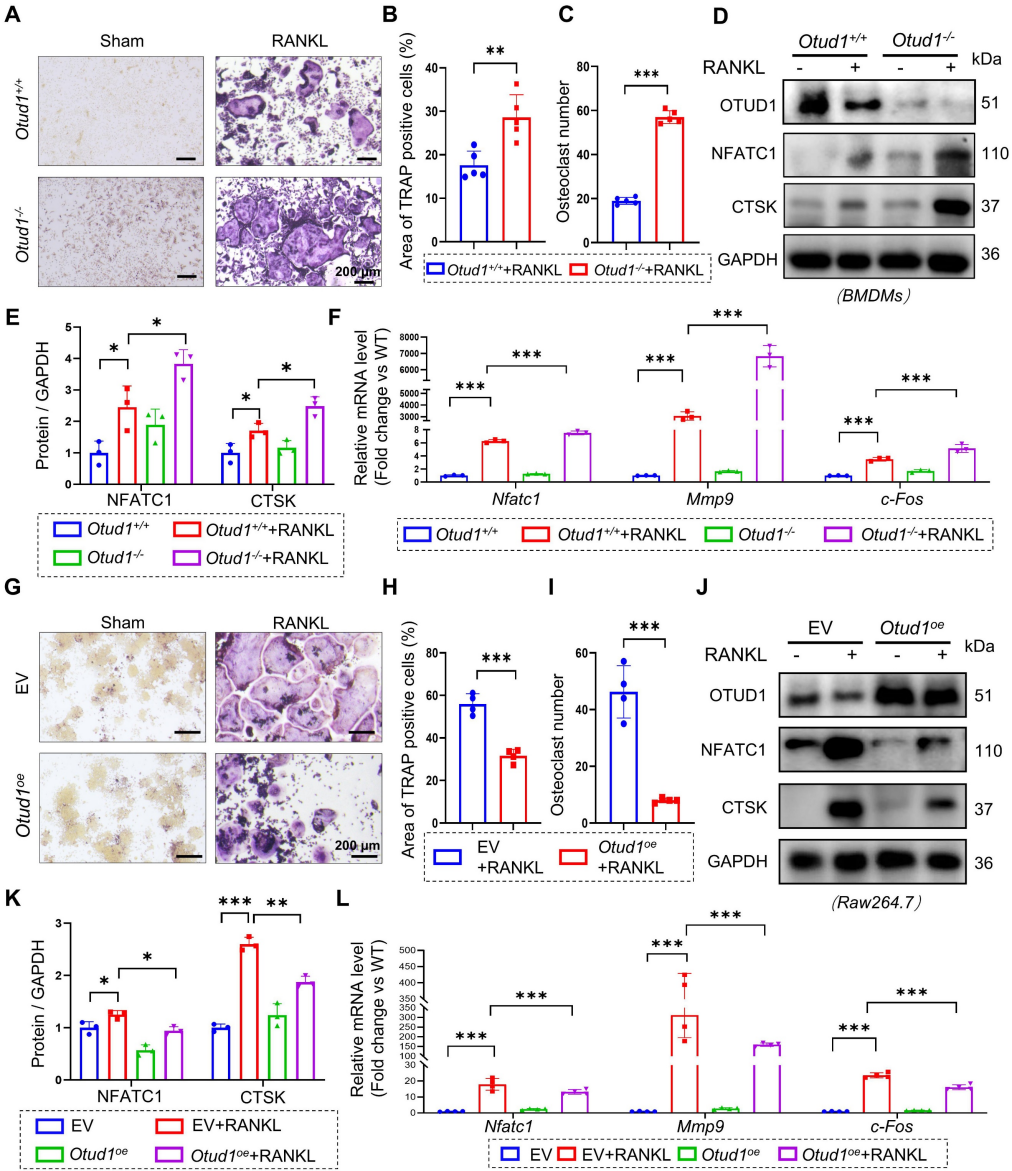
** OTUD1 suppressed osteoclastogenesis and osteoclast differentiation *in vitro.* (A-C)** Representative image of TRAP staining **(A)** and quantification analysis **(B-C)** of BMDMs from *Otud1^+/+^* and *Otud1*^-/-^ mice after 4 days of RANKL-induction (n = 4, 200 μm). **(D-E)** Representative immunoblot **(D)** and quantification **(E)** of Nfatc1 and c-Fos levels in BMDMs from *Otud1^+/+^* and *Otud1*^-/-^ mice after 4 days of RANKL-induction (n = 3). **(F)** Quantitative RT-qPCR analysis of* Nfatc1*, *Mmp9* and *c-fos* mRNA levels in BMDMs from *Otud1^+/+^* and *Otud1*^-/-^ mice after 4 days of RANKL-induction (n = 3). **(G-I)** Representative image of TRAP staining **(G)** and quantification analysis **(H-I)** of OTUD1-overexpressing and control RAW264.7 cells after 4 days of RANKL-induction (n = 4, 200 μm). **(J-K)** Representative immunoblot **(J)** and quantification** (K)** of Nfatc1 and c-Fos levels in OTUD1-overexpressing and control RAW264.7 cells after 4 days of RANKL-induction (n = 3). **(L)** Quantitative RT-qPCR analysis of *Nfatc1*, *Mmp9* and *c-fos* mRNA levels in OTUD1-overexpressing and control RAW264.7 cells after 4 days of RANKL-induction (n = 4). Data are presented as mean ± SEM. *p < 0.05, **p < 0.01, ***p < 0.001.

To delve deeper into the role of OTUD1 in the osteoclast lineage, we isolated BMDMs from the femurs of *Otud1^+/+^* and *Otud1^-/-^* mice. The results demonstrated that OTUD1 knockdown exacerbated RANKL-induced osteoclastogenesis and osteoclast differentiation, as evidenced by the increased number and area of osteoclasts (Figure 3A-C) and elevated protein and mRNA levels of bone resorption marker genes (Figure 3D-F). Conversely, OTUD1 overexpression inhibited the osteoclast differentiation of BMDMs (Figure 3G-L). Furthermore, OTUD1 deficiency did not affect the levels of p-p65 and p-p38 during osteoclast differentiation (Figure S5). Collectively, these results indicated that OTUD1 inhibited osteoclast differentiation.

### Identification of PRDX1 as a potential substrate of OTUD1

**Figure 4:**
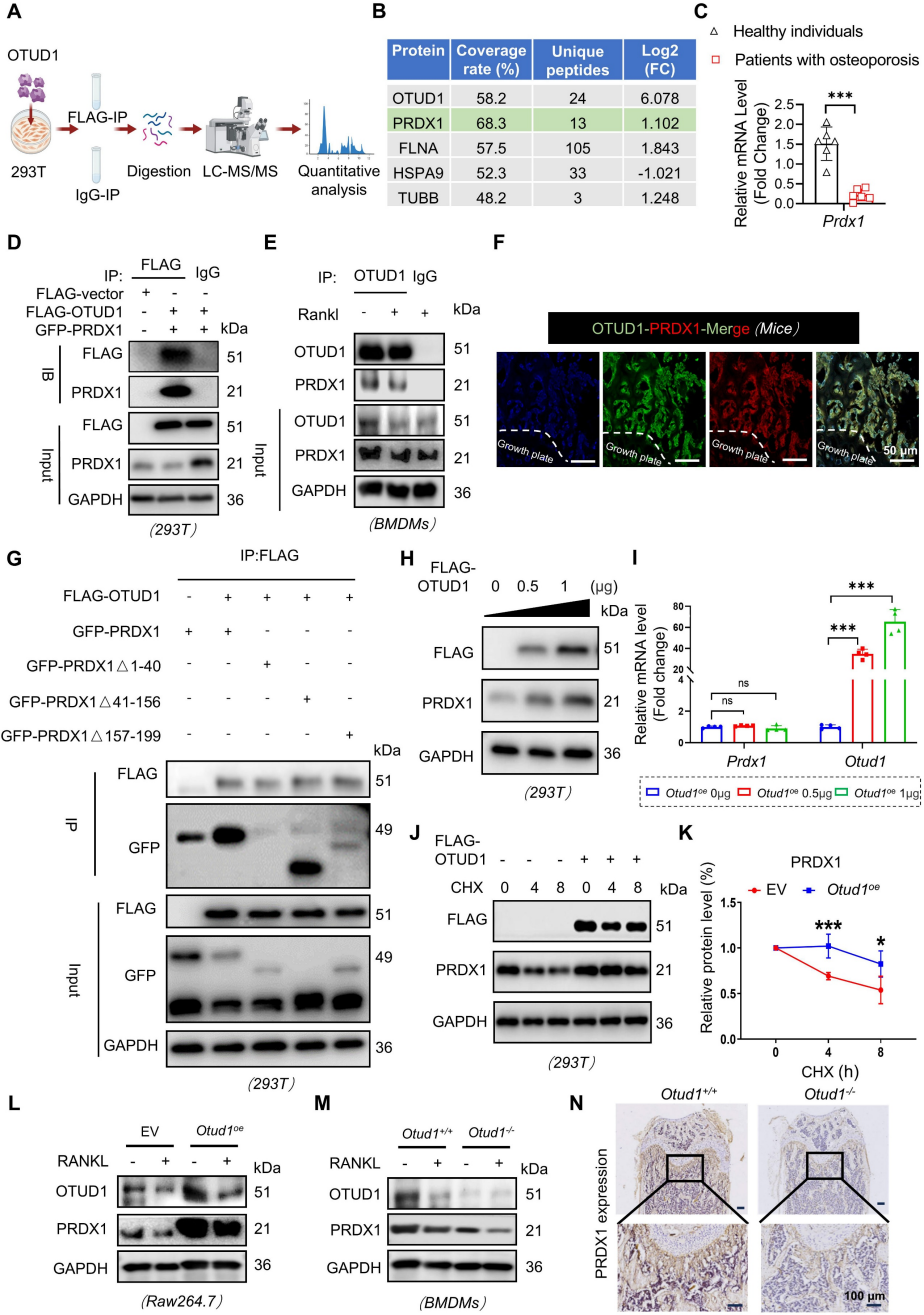
** Identification of PRDX1 as a potential substrate of OTUD1. (A)** Schematic illustration of the quantitative proteomic screen to identify proteins that bind to OTUD1. **(B)** Four potential substrates for deubiquitination of OTUD1 from liquid chromatography-tandem mass spectrometry (LC-MS/MS) analysis. (**C**) *Prdx1* mRNA levels in the femoral bone of healthy individuals and patients with osteoporosis. **(D-E)** Co-immunoprecipitation (Co-IP) of OTUD1 and PRDX1 in HEK-293T cells **(D)** and BMDMs **(E)** (n = 3). **(F)** Immunofluorescent (IF) staining showing colocalization of OTUD1 and PRDX1 in healthy mice femur (n = 3, 50 μm). **(G)** Co-IP of OTUD1, WT-PRDX1, and mut-PRDX1 in HEK-293T cells co-transfected with GFP-wt-PRDX1, GFP-mut-PRDX1 and FLAG-OTUD1 overexpression plasmids. Exogenous OTUD1 was immunoprecipitated using an anti-FLAG antibody (n = 3). **(H-I)** Representative immunoblot** (H)** and RT-qPCR** (I)** of OTUD1 and PRDX1 in HEK-293T cells transfected with overexpression plasmids of FLAG-OTUD1 (n = 3). **(J-K)** Representative immunoblot for PRDX1 in control or OTUD1-overexpressing HEK-293T cells subjected to CHX pulse-chase assay **(J)** and quantification of PRDX1 **(K)**. **(L-M)** Representative immunoblot of PRDX1 levels in RAW 264.7 cells with OTUD1 overexpression and BMDMs from *Otud1*^-/-^ mice (n = 3). **(N)** Representative images of PRDX1 staining of trabecular bone surface in distal femur from *Otud1^+/+^* and *Otud1*^-/-^ mice (n = 6, 100µm). Data are presented as mean ± SEM. *p < 0.05, ***p < 0.001, ns: no significant.

To explore the mechanism by which OTUD1 limits osteoclast differentiation, we isolated BMDMs from* Otud1^+/+^* and *Otud1*^-/-^ mice, and employed liquid chromatography-tandem mass spectrometry (LC-MS/MS) to identify potential targets of OTUD1 (Figure 4A). Intriguingly, PRDX1 emerged as a binding protein of OTUD1, distinguished by its coverage rate, number of unique peptide, and FLAG/IgG ratios (Figure 4B and Figure S6). More notably, the gene expression of OTUD1 was significantly downregulated in the femoral bone tissue of patients with osteoporosis compared to healthy controls (Figure 4C and Table S1). Subsequent Co-IP experiments confirmed the direct interaction between OTUD1 and PRDX1 in both Human Embryonic Kidney 293T (HEK-293T) cells and BMDMs (Figure 4D-E). Furthermore, immunofluorescent staining showed the colocalization of OTUD1 and PRDX1 in healthy mice femurs (Figure 4F). PRDX1 comprises three different domains: amino acids 1-40, 41-156, and 157-199 19. To pinpoint the domain of PRDX1 bound to OTUD1, we generated Flag-tagged PRDX1 truncation mutants (Figure S7A). By co-transfecting OTUD1 and mutated PRDX1 plasmids into HEK-293T cells, we confirmed that the N-terminal domain (NTD, amino acids 1-40) of PRDX1 was indispensable for its interaction with OTUD1 (Figure 4G). In summary, OTUD1 is bound specifically to the NTD of PRDX1, and PRDX1 is a potential substrate of OTUD1 during osteoclast differentiation**.**

The interaction between OTUD1 and PRDX1 prompts us to examine the regulatory functions of OTUD1 on PRDX1, including the stability and ubiquitination status. Our observations revealed that overexpression of OTUD1 elevated the protein level of PRDX1, without affecting its mRNA level (Figure 4H-I and Figure S7B). To solidify this finding, we transfected Flag-tagged OTUD1 vectors into HEK-293T cells subjected to cycloheximide (CHX) treatment. The results showed that OTUD1 hindered the degradation of PRDX1, thereby stabilizing its protein level (Figure 4J-K). Furthermore, during osteoclastogenesis, depletion of OTUD1 resulted in decreased PRDX1 protein level, while overexpression of OTUD1 led to increased PRDX1 protein level (Figure 4L-M and Figure S7C-D). *In vivo* studies further underscored that OTUD1 loss decreased the PRDX1 protein level in femur, confirming that OTUD1 maintained the PRDX1 protein level (Figure 4N and Figure S7E).

### OTUD1 reversed the K48-linked ubiquitination of PRDX1 at K16 site via C320

**Figure 5:**
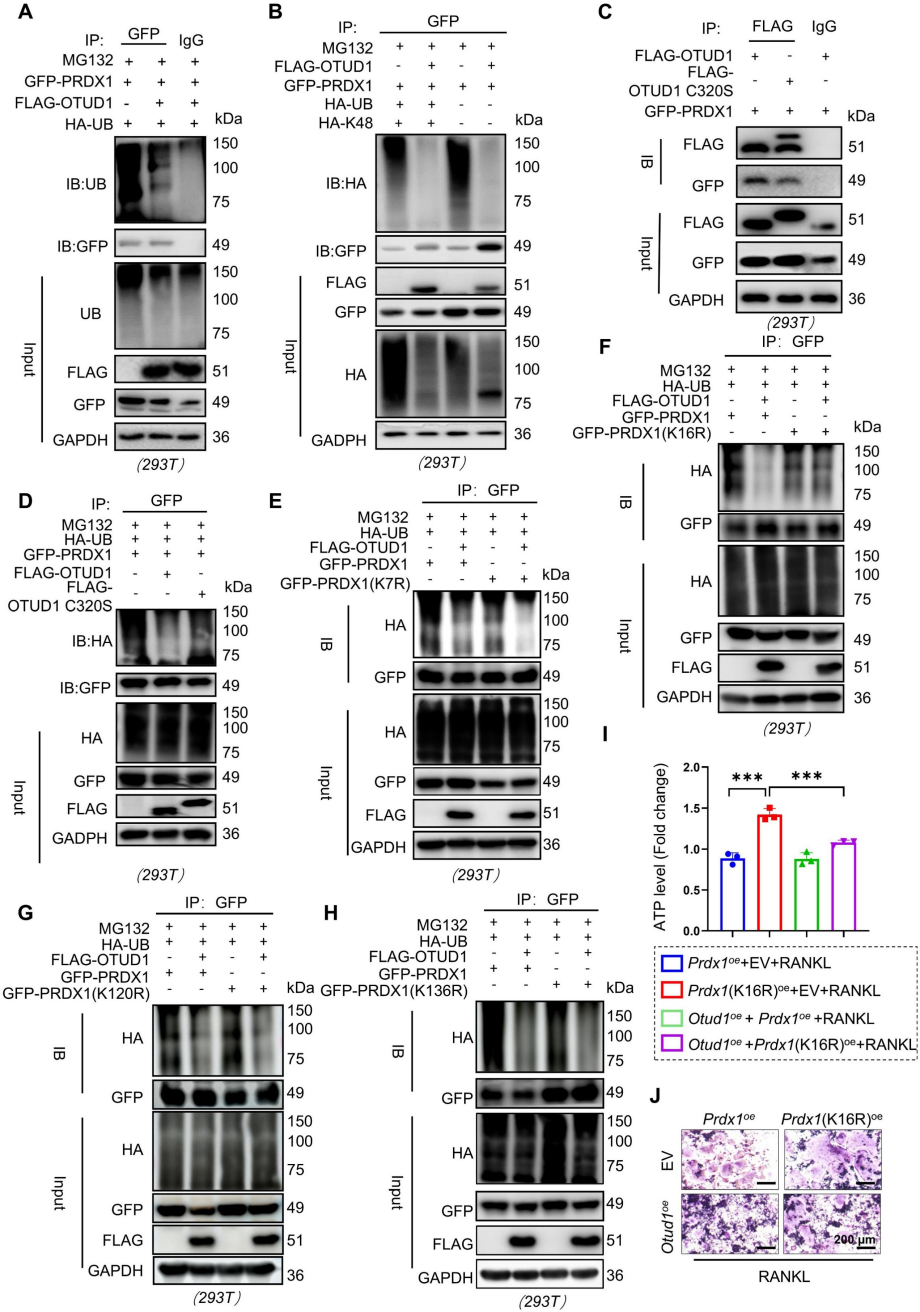
** OTUD1 reversed K48-linked ubiquitination of PRDX1via C320 of OTUD1 and removed the K16 ubiquitination of PRDX1. (A)** Results of ubiquitination assays confirming the ubiquitination of PRDX1 after overexpression of OTUD1 and PRDX1 for 24 h and treatment with MG132 (50 μM) for 6 h in HEK-293T cells (n = 3). **(B)** Immunoprecipitation of PRDX1 in 293T cells co-transfected with GFP-PRDX1, FLAG-OTUD1, and HA-Ub, HA-Ub-K48 (K48 only), and then challenged with 10 μM MG132 for 6 h. Ubiquitinated PRDX1 was detected by immunoblotting using an anti-HA antibody (n = 3). **(C)** Co-immunoprecipitation (Co-IP) of OTUD1 and PRDX1 in HEK-293T cells after overexpression of FLAG-OTUD1, FLAG-OTUD1C320S, and GFP-PRDX1 (n = 3). **(D)** Results of ubiquitination assays confirming the ubiquitination of PRDX1 after overexpression of FLAG-OTUD1, FLAG-OTUD1C320S, and GFP-PRDX1 for 24 h and treatment with MG132 (10 μM) for 6 h in HEK-293T cells (n = 3). **(E-H)** Immunoprecipitation of PRDX1 in HEK-293T cells co-transfected with FLAG-OTUD1, HA-Ub, GFP-PRDX1, GFP-PRDX1^K7R^, GFP-PRDX1^K16R^, GFP-PRDX1^K120R^, and GFP-PRDX1^K136R^ overexpression plasmids. Exogenous ubiquitinated PRDX1 was detected by immunoblotting using a GFP-specific antibody to identify the active site of OTUD1 that regulates the ubiquitination of PRDX1 (n = 3). **(I-J)** RAW 264.7 cells were co-transfected with FLAG-OTUD1, GFP-PRDX1, and GFP-PRDX1^K16R^ overexpression plasmids and stimulated with RANKL. ATP levels **(I)** and TRAP staining **(J)** of cells upon different treatment (n = 3, 200µm). Data are presented as mean ± SEM. ***p < 0.001.

HEK-293T cells were co-transfected with plasmids encoding Flag-OTUD1, GFP-PRDX1, and HA-tagged ubiquitin, followed by treatment with proteasome inhibitor MG132. The results demonstrated that OTUD1 overexpression significantly reduced PRDX1 ubiquitination (Figure 5A). Notably, K48-linked ubiquitination is a classic type of ubiquitination modification for substrate protein degradation via the 26S proteasome 24. K63 linkage typically regulates the protein-protein interactions or protein function 25. The results demonstrated that OTUD1 specifically cleaved K48-linked polyubiquitin chains from PRDX1, without altering K63-linked polyubiquitination (Figure 5B and Figure S8). Furthermore, Cysteine at 320 sites (C320) is a crucial residue in OTUD1 for its deubiquitinating enzyme activity 26. We mutated C320 to S320 to create a FLAG-OTUD1 C320S mutant plasmid. Although Flag-OTUD1 C320S bound to PRDX1 (Figure 5C), it did not deubiquitinate PRDX1 in HEK-293T cells (Figure 5D), indicating that OTUD1 reversed the ubiquitination of PRDX1 through the C320 active site.

To identify the deubiquitination sites of PRDX1, we used the GPS-Uber prediction tool 27 and identified four high-scoring lysine residues at positions 7, 16, 120, and 132. By mutating these lysine (K) residues to arginine (R) residue, we found that OTUD1 primarily removed ubiquitin molecules from K16 of PRDX1 (Figure 5E-H). In addition, transfection with the PRDX1 (K16R) mutated plasmid abolished the protective effects of OTUD1 on osteoclast differentiation (Figure 5I-J and Figure S9). These findings suggested that OTUD1 inhibited osteoclast differentiation by deubiquitinating PRDX1 at K16.

### OTUD1 inhibited oxidative stress related mitochondrial dysfunction

**Figure 6:**
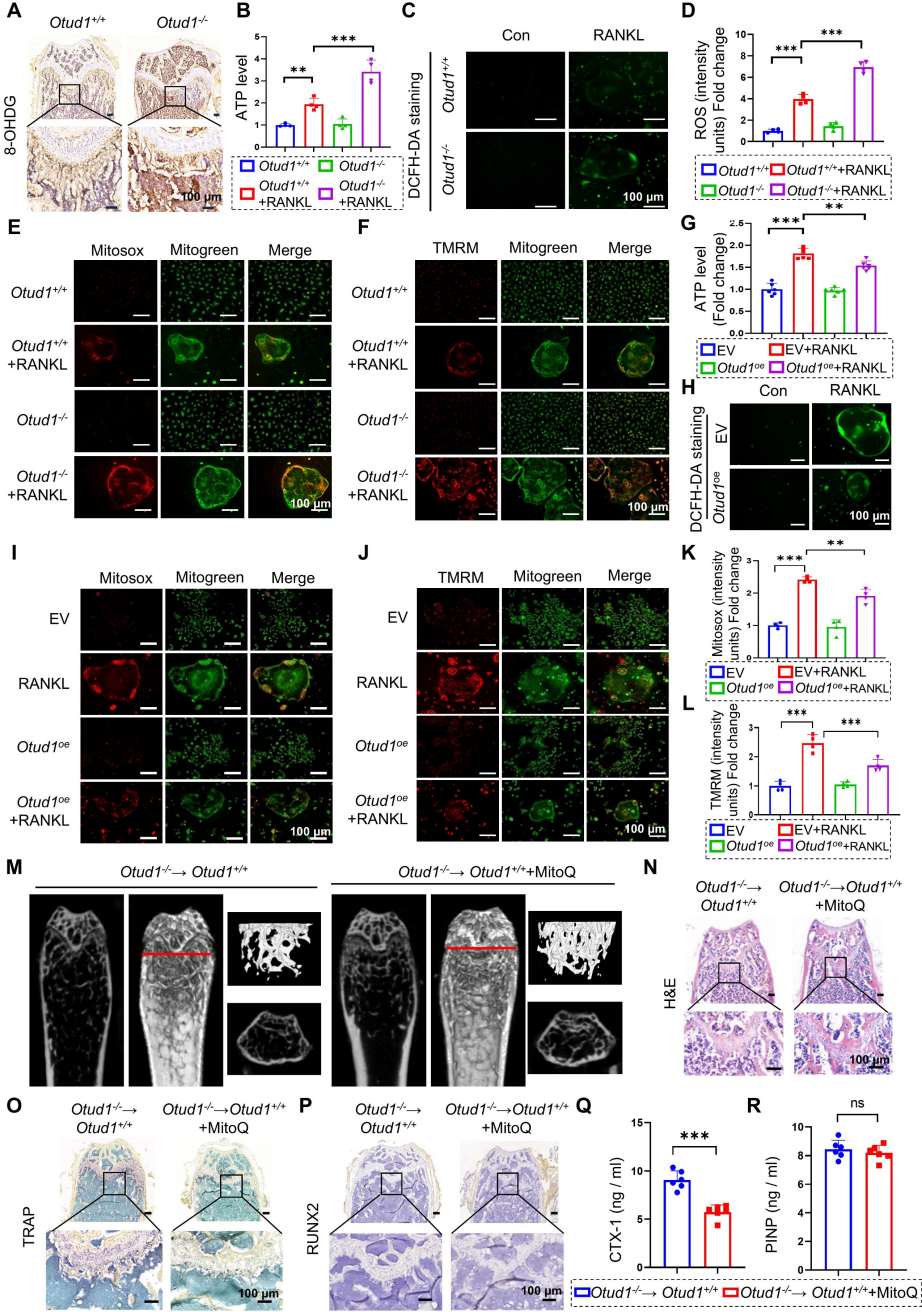
** OTUD1 regulated oxidative stress-related mitochondrial dysfunction in osteoclastogenesis. (A)** Representative image of 8-OHdG expression in femurs from *Otud1^+/+^* and *Otud1*^-/-^ mice (n = 6, 100 µm). **(B)** ATP content of BMDMs after different treatments (n = 4). **(C, D)** Representative DCFH-DA staining images **(C)** and quantitative analysis **(D)** of BMDMs after different treatments (n = 4, 100 μm). **(E-F)** Representative Mitosox and TMRM staining images of BMDMs after different treatments (n = 4, 100 μm). **(G)** ATP content of OTUD1-overexpressing and control RAW264.7 cells after different treatments (n = 6).** (H)** Representative DCFH-DA staining images of OTUD1-overexpressing and control RAW264.7 cells after different treatments (n = 4, 100 μm). **(I-L)** Representative Mitosox and TMRM staining images and quantitative analysis of OTUD1-overexpressing and control RAW264.7 cells after different treatments (n = 4, 100 μm).** (M)** Representative micro-CT images of the distal trabecular bone of femurs from *Otud1^+/+^* mice receiving *Otud1*^-/-^ bone marrow treated with or without MitoQ (n = 6). **(N)** H&E staining of femurs from* Otud1^+/+^* mice receiving *Otud1*^-/-^ bone marrow treated with or without MitoQ, with the boxed area in the first panel magnified below (n = 6, 100 μm). **(O)** Representative image of TRAP staining of distal trabecular and cortical bone of femurs from* Otud1^+/+^* mice receiving *Otud1*^-/-^ bone marrow treated with or without MitoQ, with the boxed area in the first panel magnified below (n = 6, 100 μm). **(P)** Immunohistochemical staining of RUNX2 in femurs from *Otud1^+/+^* mice receiving *Otud1*^-/-^ bone marrow treated with or without MitoQ, with the boxed area in the first panel magnified below (n = 6, 100 μm). **(Q-R)** ELISA measurements of CTX-1 **(Q)** and PINP** (R)** levels in the serum of* Otud1^+/+^* mice receiving *Otud1*^-/-^ bone marrow treated with or without MitoQ (n = 6). Data are presented as mean ± SEM. **p < 0.01, ***p< 0.001, ns: no significant.

PRDX1 is a pivotal peroxidase that protects cells from oxidative stress damage 28. Numerous studies have revealed that oxidative stress is closely associated with mitochondrial dysfunction. PRDX1 plays a significant role in maintaining mitochondrial morphology and function 29. Given the essential role of PRDX1 in mitochondrial integrity 34, we characterized its function in oxidative stress mitigation and mitochondrial protection within the OTUD1-PRDX1 regulatory pathway. Our findings revealed that the protein level of 8-hydroxy-2 deoxyguanosine (8-OHDG), a vital biomarker of oxidative damage, were elevated in the femurs of *Otud1*^-/-^ mice compared to those in *Otud1^+/+^* littermates (Figure 6A). Furthermore, OTUD1 deficiency altered the gene expression of genes associated with mitochondrial function, leading to a notable upregulation of *Pgc1-α* and downregulation of *Nd1*, *Nd2*, and* Nd4* in the femurs of mice (Figure S10A-D). Remarkably, ablation of OTUD1 exacerbated mitochondrial dysfunction during osteoclastogenesis, as evidenced by increased ATP, total ROS, mtROS, and mitochondrial membrane potential (MMP) levels (Figure 6B-F, and Figure S10E-F). Conversely, OTUD1 overexpression reduced ATP, total ROS levels, mtROS, and MMP level during osteoclastogenesis (Figure 6G-L, and Figure S10G). Interestingly, RANKL stimulation induced mitochondrial fragmentation in osteoclasts, characterized by smaller mitochondria with diminished cristae structures, which was reversed by OTUD1 overexpression and enhanced by OTUD1 knockdown (Figure S10H-I). Furthermore, MitoQ alleviated osteoclastogenesis and mitochondrial dysfunction exacerbated by OTUD1 deficiency (Figure S11A-H). To further validate the role of OTUD1-mitochondrial function axis in regulating bone metabolism, we used MitoQ in myeloid cell-specific OTUD1-deficient mice. The *in vivo* results demonstrated that MitoQ alleviated bone loss and reduced the number and size of TRAP-positive osteoclasts in myeloid cell-specific OTUD1-deficient mice (Figure 6M-O and Figure S11I-L). Immunohistochemical staining revealed no significant differences in RUNX2 levels between the two groups (Figure 6P). Furthermore, MitoQ decreased the serum levels of CTX-1 in myeloid cell-specific OTUD1-deficient mice, whereas PINP levels remained unaffected (Figure 6Q-R). In summary, OTUD1 inhibited osteoclastogenesis by amelioration of oxidative stress related mitochondrial dysfunction.

### OTUD1-PRDX1-mitochondrial function axis played a pivotal role in regulating bone metabolism

**Figure 7:**
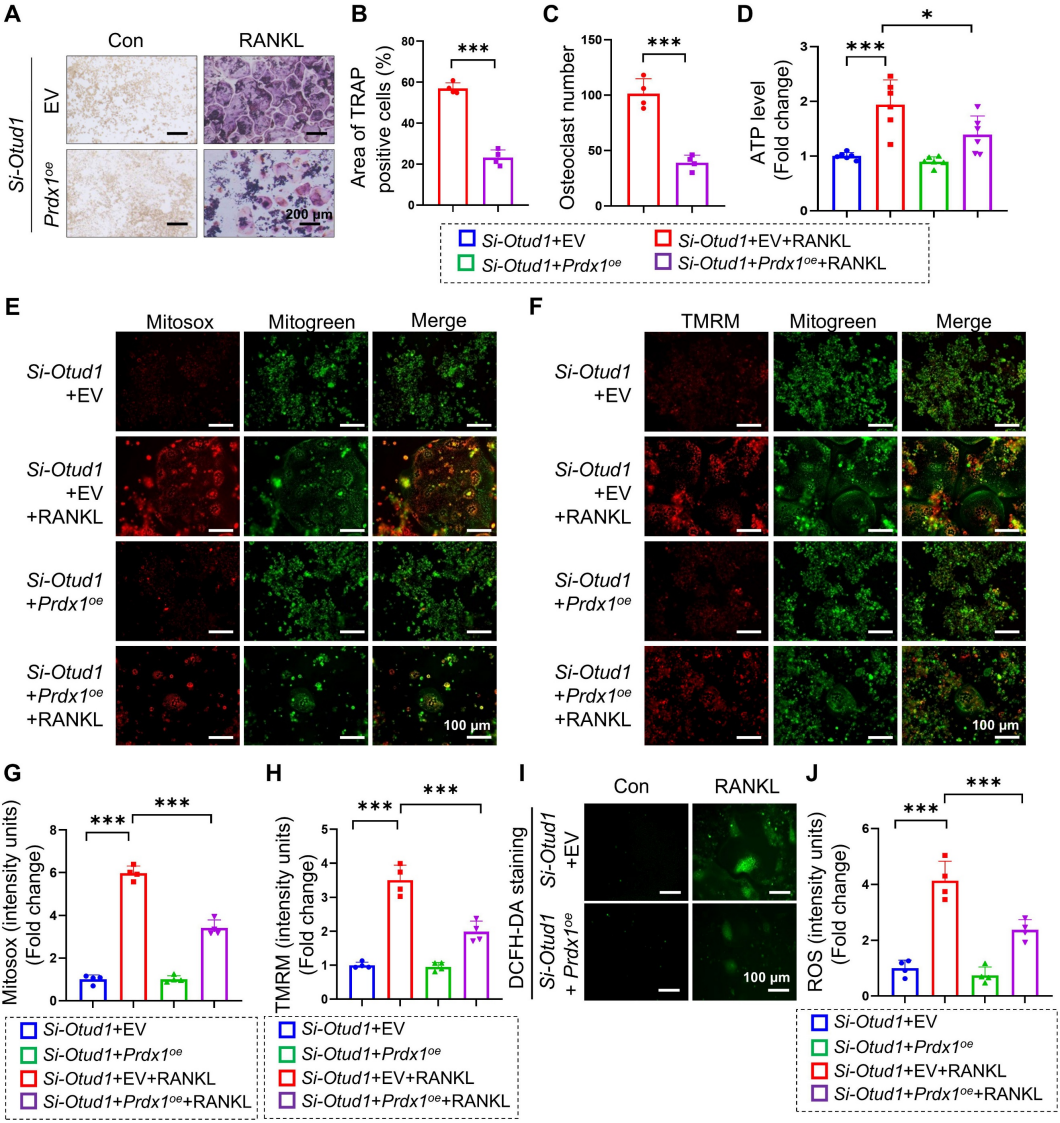
** PRDX1 rescued OTUD1 deficiency-induced bone mass loss and mitochondrial dysfunction. (A-C)** Representative image of TRAP staining **(A)** and quantification analysis **(B-C)** of RAW264.7 cells transfected with or without *Prdx1^oe^* (or EV) and si-OTUD1 after RANKL-induction (n = 4, 200 μm). **(D)** ATP content of RAW264.7 cells transfected with *Prdx1^oe^* (or EV) and si-OTUD1 after RANKL-induction (n = 6). **(E-H)** Representative Mitosox staining images **(E)** and quantitative analysis** (G)** of RAW264.7 cells transfected with *Prdx1^oe^* (or EV) and si-OTUD1 after 4 days of RANKL-induction (n = 4, 100 μm). Representative TMRM staining images **(F)** and quantitative analysis **(H)** of RAW264.7 cells transfected with *Prdx1^oe^* (or EV) and si-OTUD1 after 4 days of RANKL-induction (n = 4, 100 μm). **(I-J)** Representative DCFH-DA staining images **(I)** and quantitative analysis **(J)** of RAW264.7 cells transfected with *Prdx1^oe^* (or EV) and si-OTUD1 after RANKL-induction (n = 4, 100 μm). Data are presented as mean ± SEM. *p < 0.05, ***p < 0.001.

We subsequently investigated whether PRDX1 mediates the regulation of OTUD1 during osteoclastogenesis. Our results revealed that the protein levels of PRDX1 were progressively downregulated during osteoclastogenesis (Figure S12A-B). Furthermore, overexpression of PRDX1 overexpression inhibited osteoclast differentiation and related mitochondrial dysfunction (Figure S12C-E), manifested by decreased ATP production, total ROS levels, mtROS levels, and MMP levels in BMDMs (Figure S12F-L). Moreover, PRDX1 overexpression reduced the protein expression of osteoclastogenic markers, including NFATC1 and CTSK (Figure S12M-N). We further examined whether PRDX1 is involved in osteoclastic differentiation enhanced by OTUD1 deficiency. The results showed that PRDX1 overexpression attenuated osteoclastogenesis and osteoclast differentiation triggered by OTUD1 loss (Figure 7A-C). Notably, PRDX1 overexpression attenuated mitochondria dysfunction in OTUD1-deficient BMDMs, as evidenced by decreased ATP production, mtROS and MMP level, and total ROS level (Figure 7D-J). These data indicated that PRDX1 rescued osteoclastogenesis and mitochondrial dysfunction enhanced by OTUD1 deficiency, highlighting the critical regulatory role of the OTUD1-PRDX1 axis in osteoclastogenesis.

The effects of osteoclasts can vary under different physiological and pathological conditions 1. Inflammatory stimuli promote bone resorption, leading to overt bone loss in patients in both clinical and animal models 30, 31. Given the key regulatory role of OTUD1 in bone metabolism, it is necessary to elucidate its specific role in bone metabolism under pathological conditions. We established a calvarial osteolysis model by injecting LPS around the sagittal suture of the skull in *Otud1^+/+^* and *Otud1*^-/-^ mice. The results showed that the whole-body OTUD1 knockout aggravated LPS-induced calvaria destruction, as reflected by wider sutures, decreased BV/TV volume, and increased number of osteoclasts (Figure S13). Furthermore, we explored the extent to which OTUD1 contributes to bone homeostasis via the PRDX1-mitochondrial dysfunction axis. The results showed that PRDX1 knockdown diminished the protective effect of OTUD1 against osteoclastogenesis by increasing the number of osteoclasts and TRAP-positive areas (Figure S14).

## Discussion

Bone diseases, characterized by inadequate bone formation and excessive bone resorption, are highly prevalent and cause tremendous damage over long treatment cycles 32. The conspicuous absence of effective treatments for bone diseases poses a significant and escalating challenge to public health 3, 33. Osteoclasts mediate bone resorption, making their differentiation and activation key therapeutic targets for bone disorders 1. Previous studies have implicated OTUD1 in multiple pathological conditions, including inflammatory bowel disease 14, hepatic ischemia/reperfusion injury 34, and hypertensive cardiac hypertrophy 26. However, the role of OTUD1 in bone metabolism remains unclear. Here, we provide compelling* in vivo* and* in vitro* evidence that OTUD1 deficiency in myeloid cells induced bone loss and highlighted OTUD1 as a new and critical regulator of osteoclast-related bone metabolism. Mechanistically, OTUD1 preserved the stability of PRDX1 by reversing K48-linked ubiquitination of PRDX1, which mitigated oxidative stress related mitochondrial dysfunction and ultimately inhibited osteoclast differentiation. These findings suggest that OTUD1 is a potential therapeutic target for osteoclast-dependent bone diseases.

Accumulating evidence has consistently highlighted the pivotal role of protein ubiquitination in bone metabolism. E3 ubiquitin ligases, notably Smurf1, Smurf2, and RNF146, played crucial roles in osteoblast differentiation and bone formation processes 35. DUBs, such as USP7 9, USP34 36, and USP53 37, have been identified as critical regulators of bone remodeling through their modulation of osteoblasts, osteoclasts, and osteocytes differentiation and function. However, the development of potential drugs targeting these E3 ubiquitin ligases and DUBs for the therapeutic management of bone disorders requires further investigation 35. Our study identified OTUD1 as a novel regulator of bone metabolism, that protects against bone loss. OTUD1 deficiency exacerbated bone loss primarily through increased bone resorption, rather than impaired bone formation. Notably, OTUD1 in myeloid cells contributed to bone loss. OTUD1 was primarily expressed in the trabecular bone, bone marrow cavity, and periosteum of adult mice, with few OTUD1^+^ cells present in the growth plates. Consistent with this finding, neither whole-body knockout nor myeloid-specific deficiency of OTUD1 affected bone size because of the limited expression of OTUD1 in the growth plate. Considering the important role of OTUD1 in regulating osteoclast differentiation, we speculated that OTUD1 may regulate osteoclastogenesis in multiple bone metabolic diseases, which warrants further validation with clinical studies. Currently, no small molecule activators of OTUD1 have been identified. Future studies should employ OTUD1-overexpressing adenoviruses or develop OTUD1-targeted agonists to evaluate therapeutic potential in animal models and clinical trials. Notably, OTUD1 exhibited context-dependent duality, with both protective and detrimental effects across different cells 25, 38. This suggests systemic OTUD1 activation risks off-target effects, necessitating bone-specific delivery systems for OTUD1 protein or gene therapy. A recent study developed protein fate-regulating functional microunits (PFFMs) comprising GelMA microspheres with USP26-overexpressing BMSCs in polycaprolactone scaffolds, which enhanced rat intervertebral fusion. This PFFM system may provide promise for delivering osteoclast-specific OTUD1 therapies in the future 5.

DUBs regulate protein stability by removing ubiquitin from target proteins. PRDX1 was identified as a potential substrate of OTUD1 using LC-MS/MS and Co-IP analysis. As a key 2-Cys antioxidant enzyme, PRDX1 maintains redox homeostasis by scavenging ROS, and counteracting mitochondrial dysfunction 18. Although PRDX1 is recognized as a regulator of bone metabolism, its molecular mechanisms remain elusive. Current evidence demonstrated that PRDX1 played dual roles in bone metabolism: suppressing osteoclastogenesis through ROS/NFATc1 inhibition 39, while paradoxically promoting osteoblast proliferation yet blocking their differentiation 40. Furthermore, a study confirmed that PRDX1 deficiency exacerbated osteoclast differentiation and bone loss 41. Despite these studies, the precise regulatory mechanisms of PRDX1 in bone homeostasis, particularly its post-translational modifications and downstream pathways, require further elucidation. Our study confirmed that PRDX1 overexpression abolished osteoclastogenesis enhanced by OTUD1 deficiency. Furthermore, it is well established that mitochondrial oxidative stress and dysfunction critically regulate bone metabolism and osteoclast differentiation 42, 43. Our experiments revealed that OTUD1 ablation aggravated oxidative damage and mitochondrial dysfunction, which were rescued by MitoQ. These results established that OTUD1 was essential for maintaining proper mitochondrial function in osteoclasts. Collectively, our work identified the OTUD1-PRDX1-mitochondrial axis as a key regulatory pathway in osteoclastogenesis, suggesting novel therapeutic opportunities for bone disorders through targeted modulation of this axis.

Although PRDX1 was previously thought to be exclusively cytoplasmic, recent microscopic evidence has demonstrated its widespread distribution across the nucleus, cytoplasm, mitochondria, and peroxisomes 44-46. Further research is needed to ascertain whether OTUD1 influences PRDX1 trafficking in these cellular compartments and to elucidate the compartment-specific functions of PRDX1 in bone diseases. In addition, PRDX1 is widely expressed in various human cells and tissues 47, and agents directly targeting PRDX1 may lack specificity for bone disease and cause adverse effects. Therefore, strategies to enhance PRDX1 activity, specifically in bone tissue, such as bone-targeted delivery systems, could maximize therapeutic benefits while minimizing systemic side effects, offering a promising avenue for the treatment of osteoporosis and other bone-related disorders. Despite the well-documented osteoprotective properties of PRDX1, the current lack of direct pharmacological activators severely restricts its clinical applicability. OTUD1 represents an ideal upstream target for indirectly enhancing PRDX1 activity in a bone-selective manner for treating osteoporosis and other bone-related diseases.

Previous studies have shown that PRDX1 is modulated at both transcriptional and post-transcriptional levels 48. Studies have also indicated that PRDX1 activity may be modulated by protein modifications, including ubiquitination, acetylation, phosphorylation, glutathionylation, and S-nitrosylation 44-46. We meticulously delineated the intricate mechanism by which OTUD1 removed K48-linked ubiquitination of PRDX1. Our study established that OTUD1 specifically bound to the NTD of PRDX1. The active site C320 of OTUD1 was responsible for cleaving ubiquitin moieties from PRDX1. Furthermore, we found that OTUD1 inhibited the ubiquitination of PRDX1 at K16. A recent study revealed that PRDX1 could be acetylated at the K16 site 49. Further investigation is needed to determine whether acetylation and ubiquitination at K16 act synergistically to modulate the stability and function of PRDX1. Although OTUD1 plays a crucial regulatory role in PRDX1 modulation, it is not the only deubiquitinase that controls PRDX1. Previous studies have confirmed that the E3 ligase HECTD3 mediated the ubiquitination and degradation of PRDX1 50, whereas the E3 ligase TRIM16 inhibited PRDX1 phosphorylation 51. Whether additional members of the ubiquitin/deubiquitinase family participate in PRDX1 regulation of bone metabolism remains unclear.

Although this study provides valuable insights, it has some limitations. First, future studies should use osteoclast-specific OTUD1-deficient mice to validate the regulatory role of osteoclast OTUD1 on bone metabolism. Second, this experiment was limited to male mice to minimize potential confounding effects of estrogen fluctuations. Given the established sex differences in bone formation and resorption 52, subsequent studies should include female mice to enable a more comprehensive assessment of bone-related phenotypes. Finally, Previous studies have shown that PRDX1 inhibited osteoblast differentiation in MC3T3-E1 cells 40, whereas our study using BMSCs revealed that OTUD1, an upstream regulator of PRDX1, primarily regulated osteoclast function without affecting osteoblast differentiation. This discrepancy probably stems from cell type specificity.

In summary, our study indicated a pivotal OTUD1-PRDX1-mitochondrial dysfunction axis that maintained osteoclast homeostasis. Our findings provided the first evidence that OTUD1 suppressed osteoclastogenesis during bone metabolism by deubiquitinating PRDX1 and maintaining PRDX1 stability, thereby offering a promising therapeutic approach for alleviating osteoclast-dependent bone diseases.

## Supplementary Material

Supplementary figures and tables.

## Abbreviations

DUBs: deubiquitinating enzymes
OTUD1: ovarian tumor deubiquitinase 1
LC-MS/MS: liquid chromatography-tandem mass spectrometry
Co-IP: co-immunoprecipitation
PRDX1: peroxiredoxin 1
MitoQ: mitochondria-targeted ubiquinone
UPS: ubiquitin-proteasome system
USPs: ubiquitin-specific proteases
OTUDs: ovarian tumor proteases
ROS: reactive oxygen species
ECCR: ethics committee in clinical research
LPS: lipopolysaccharide
Micro-CT: micro-computed tomography
PFA: paraformaldehyde
ROI: region of interest
BV/TV: trabecular bone volume per tissue volume
Tb. Th: trabecular thickness
Tb. N: trabecular number
Tb. sp: trabecular spacing
H&E: hematoxylin and eosin
TRAP: tartrate-resistant acid phosphatase
OCT: Optimal cutting temperature
DAPI: 4',6-Diamidino-2-Phenylindole
CTX-1: collagen type 1
PINP: type I procollagen
BMDMs: bone marrow-derived macrophages
M-CSF: macrophage colony-stimulating factor
FBS: fetal bovine serum
BMSCs: bone marrow stromal cells
siRNA: small interfering RNA
RANKL: receptor activator of nuclear factor κB ligand
DMSO: dimethyl sulfoxide
SDS-PAGE: sodium dodecyl sulfate polyacrylamide gel electrophoresis
ALP: alkaline phosphatase
RT-qPCR: real-time quantitative polymerase chain reaction
TMRM: tetramethylrhodamine methyl Ester
MMP: mitochondrial membrane potential
mtROS: mitochondrial reactive oxygen species
ATP: adenosine triphosphate
SEM: standard error of the mean
ANOVA: analysis of variance
CTSK: cathepsin K
BMD: bone mineral density
Nfatc1: nuclear factor of activated T cells 1
RUNX2: runt-related transcription factor-2
HEK-293T: human embryonic kidney 293T
CHX: cycloheximide
C320: cysteine at 320 sites
8-OHDG: 8-hydroxy-2 deoxyguanosine
PFFMs: protein fate-regulating functional microunits
