# Supplementary material for: OTUD1 inhibits osteoclast differentiation and osteoclastic bone loss through deubiquitinating and stabilizing PRDX1
Source: Theranostics. 2025 Jun 9;15(14):6719–36. doi: 10.7150/thno.111360 (PMC12203675; doi:10.7150/thno.111360)
Supplement: Supplementary file 1 — Supplementary figures and tables. [file thnov15p6719s1.pdf]

Supplementary Information

OTUD1 inhibits osteoclast differentiation and osteoclastic bone loss through deubiquitinating and stabilizing PRDX1

Xiaoyu Sun[1,2,#], Tong Wu[1,#], Shuhong Chen[1,#], Zheyu Zhao[1], Ruiwei Jia[1],Jun Ma[1], Lei Yin[1], Xingbei Pan[1], Yifan Ping[1], Lulu Ma[1], Yilin Ma[1], Wu Luo[3], Shengbin Huang[1,4]*, Guang Liang [1,3,5]*.*

The supplementary file includes 3 tables and 14 figures.

Table S1 Clinical data from human subjects with or without osteoporosis

**Healthy individuals**					**Patients with osteoporosis**				
**ID**	**Age**	**Sex**	**X ray image**	**BMD *T*-score**	**ID**	**Age**	**Sex**	**X ray image**	**BMD *T*-score**
1	76	M	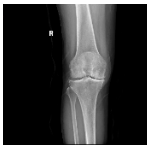	-0.8	5	78	F	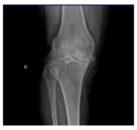	-2.8
2	74	F	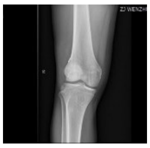	-0.6	3	75	F	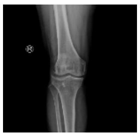	-3.3
3	72	F	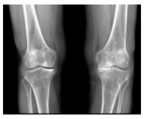	-0.8	4	71	M	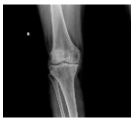	-2.6
4	69	M	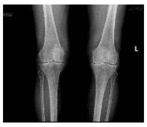	-0.7	2	62	M	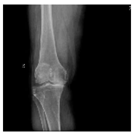	-3.1
5	55	M	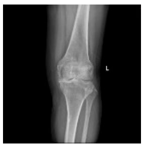	-0.9	6	54	M	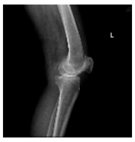	-2.9
6	52	M	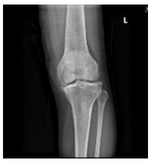	-0.6	1	50	M	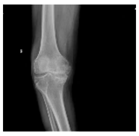	-2.5

Table S2. Primer sequences for RT-PCR assay.

**Gene (Mouse)**	**Forward Primer (5' to 3')**	**Reverse Primer (5' to 3')**
Mmp9	TCGGGAGAGAGAGGAGTCTG	CTACTGGGCGTTAGGGACAG
Nfatc1	TCCTGTCCAACACCAAAGTC	TTCCTCCCGATGTCTGTCTC
c-Fos	AGCGAGCAACTGA GAAGCC	CGCTGTGAAGCA GAGCTGG
ALP	CCAACTCTTTTGTGCCAGAGA	GGCTACATTGGTGTTGAGCTTTT
OCN	CAAAGGTGCAGCCTTTGTGTC	TCACAGTCCGGATTGAGCTCA
PGC1-α	TATGGAGTGACATAGAGTGTGCT	CCACTTCAATCCACCCAGAAAG
ND1	CAACCATTTGCAGACGCCAT	GGGTGTGGTATTGGTAGGGG
ND2	CCTATCACCCTTGCCATCATCT	GCTGCTTCAGTTGATCGTGG
ND4	ACCCGATGAGGGAACCAAAC	AGCGTCTAAGGTGTGTGTTGT
Actin	CTCCTGCCCAGACACGATG	GGACCGTCTTCTCGATGAGC
OTUB1	GCCCTCAGTGTGTCCATCCAAG	TCGTAGTGTCCAGGTCGGTAGAG
OTUB2	ACCTCATTCCTCGCTTCCATCTG	AGTGGGTAAGACAAGACGGAGAAC
OTUD1	CTCTGCCTGGCTGCTGGAAG	GGTGCTCGCTCAGTCGGAAG
OTUD3	CGGCTGCGAAGAAGAGTTTGTG	TGGCGATGCTTGAGATGGTTCC
OTUD4	ACTCCTGCGGTGCCTTCTTTAC	CGGCAGCATCAGGTCCAGTG
OTUD5	TGCCCAAACCATTCCGTACTGAG	TGCCTTTCTCCAGACTCTCCAAAC
OTUD6A	TGTCACCAACGCTCCAAGTCTG	TTGCTTGTCCCTGCTCTGTCTC
OTUD7A	ACAGCAGAACAAGGAGGAGGAATG	GAGTTGTCCACACCACCACCTG
OTUD7B	CACCAACGAAGAGGAGGAGTACAG	GGGCAAACAAGAGCACAGAGAAG
OTULIN	CACCAACGAAGAGGAGGAGTACAG	GGGCAAACAAGAGCACAGAGAAG
ALG13	TGCCCAAACCATTCCGTACTGAG	TGCCTTTCTCCAGACTCTCCAAAC
TNFAIP3	TGTCACCAACGCTCCAAGTCTG	TTGCTTGTCCCTGCTCTGTCTC
ZRANB1	AGGTGACTTAGCAGCCATAGAAGC	AGCAGAAGGACGGTTCAGCAAG

Table S3. The potential active sites of PRDX1.

Position	Peptide	Score
120	RTIAODYGVLKADEGISFRGL	0.7077
7	****MSSGNAKIGYPAPNEKA	0.6117
136	SPRGLPIIDDKGILRQITIND	0.4502
16	AKIGYPAPNEKATAVMPDGQE	0.4109

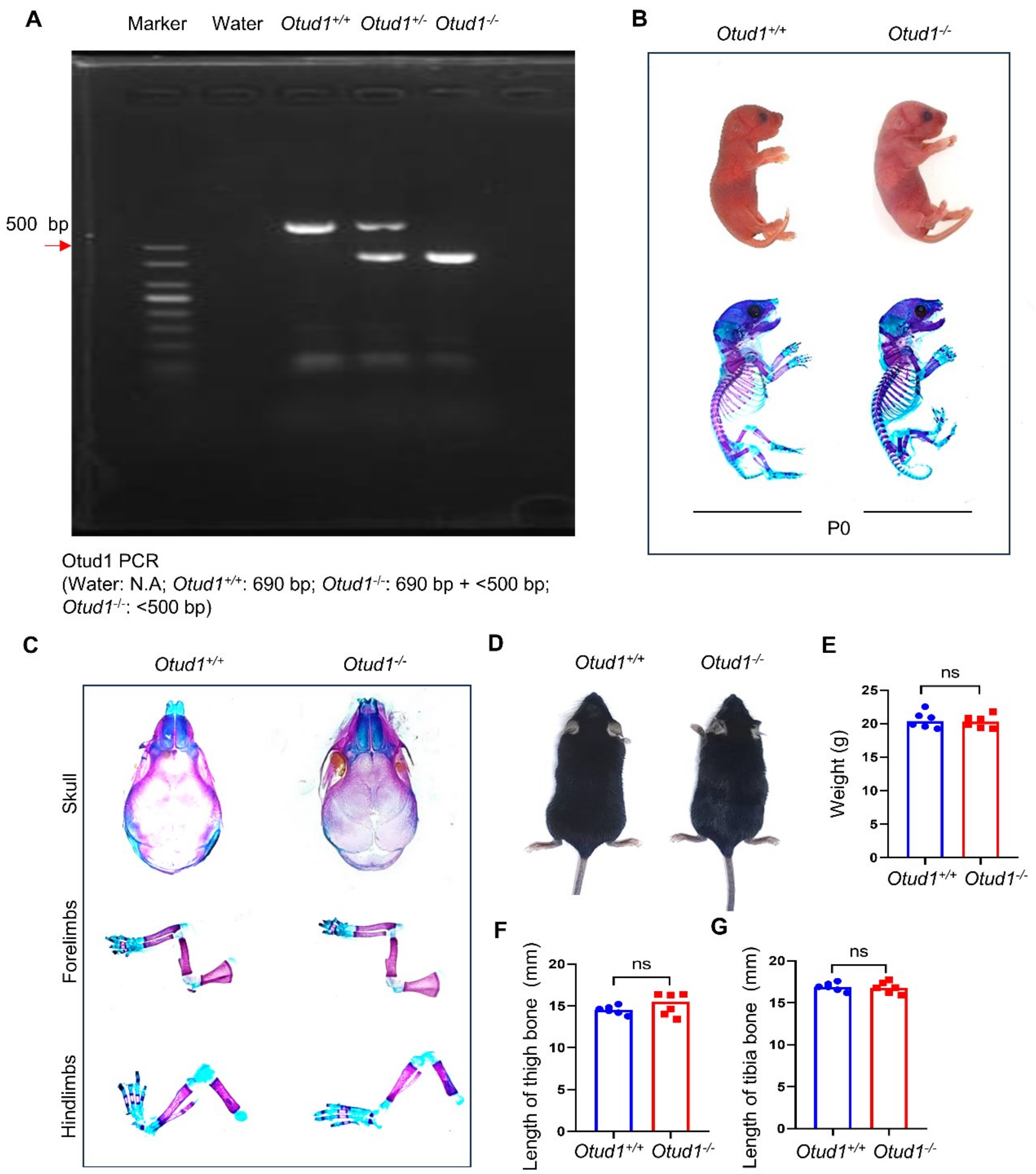

Figure S1. The general phenotypes of *Otud1*$^{+/+}$ and *Otud1*$^{-/-}$ mice.

(A) Validation of *Otud1*$^{-/-}$ mice by RT-PCR. **(B)** Representative appearance and whole skeleton of *Otud1*$^{+/+}$ and *Otud1*$^{-/-}$ embryos at postnatal (P) day P0 (*n* = 6). **(C)** Representative images of skeleton of skulls, forelimbs, and hindlimbs in *Otud1*$^{+/+}$ and *Otud1*$^{-/-}$ P0 embryos. **(D, E)** Representative image **(D)** and weight quantification **(E)** of *Otud1*$^{+/+}$ and *Otud1*$^{-/-}$ mice of 8-week (*n* = 6). **(F, G)** The femur length **(F)** and tibia length **(G)** quantification between *Otud1*$^{+/+}$ and *Otud1*$^{-/-}$ mice (*n* = 6). Data are presented as the mean ± SEM. ns: no significant.

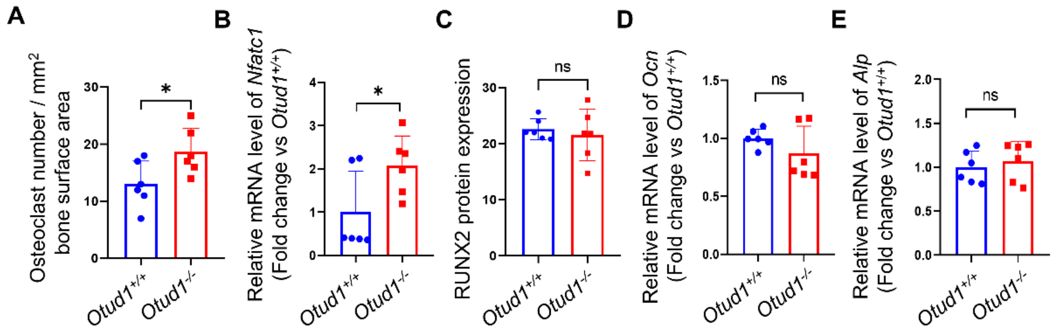

Figure S2. Impact of OTUD1 deficiency on femoral bone metabolism.

(A) Quantification analysis of number of osteoclasts in femurs from 8-week-old *Otud1*$^{+/+}$ and *Otud1*$^{-/-}$ mice (n = 6). **(B)** Quantitative RT-PCR analysis of *Nfatc1* in femur tissues from *Otud1*$^{+/+}$ and *Otud1*$^{-/-}$ mice (n = 6). **(C)** Immunohistochemistry staining of RUNX2 in femurs from 8-week-old *Otud1*$^{+/+}$ and *Otud1*$^{-/-}$ mice (n = 6). **(D, E)** Quantitative RT-PCR analysis of osteogenesis genes including *Ocn* **(D)** and *Alp* **(E)** mRNA levels in femur tissues from *Otud1*$^{+/+}$ and *Otud1*$^{-/-}$ mice (n = 6). Data are presented as the mean ± SEM. *$p < 0.05$, ns: no significant.

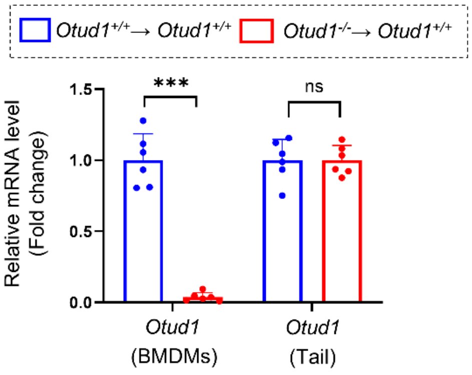

Figure S3. Efficiency of myeloid-specific knockdown of OTUD1.

RT-qPCR analysis of *Otud1* levels in bone marrow-derived macrophages (BMDMs) from femur bones and tail tissues of the *Otud1*$^{+/+}$→*Otud1*$^{+/+}$ and *Otud1*$^{-/-}$→*Otud1*$^{+/+}$ mice (n = 6). ***$p < 0.001$, ns: no significant.

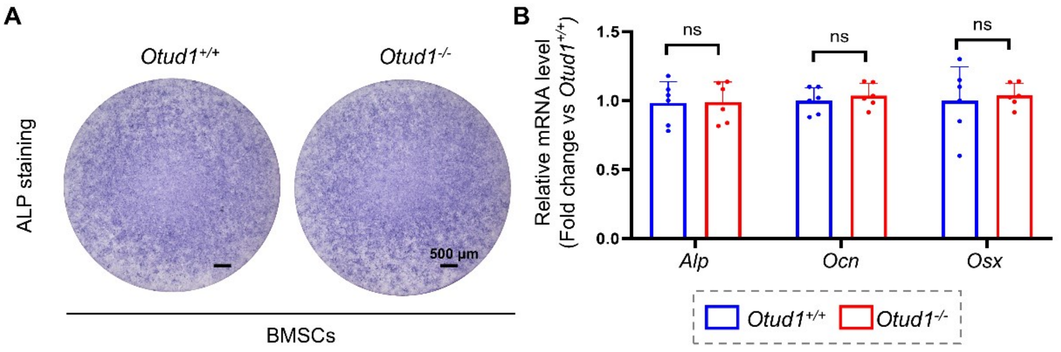

Figure S4. Osteogenic differentiation of BMSCs from *Otud1*$^{+/+}$ and *Otud1*$^{-/-}$ mice.

(A) Representative image of ALP staining of BMSCs from *Otud1*$^{+/+}$ and *Otud1*$^{-/-}$ mice after osteogenic differentiation (n = 3, 500 μm). **(B)** Quantitative RT-PCR analysis of osteogenesis genes mRNA levels in BMSCs from *Otud1*$^{+/+}$ and *Otud1*$^{-/-}$ mice (n = 8). ns: no significant.

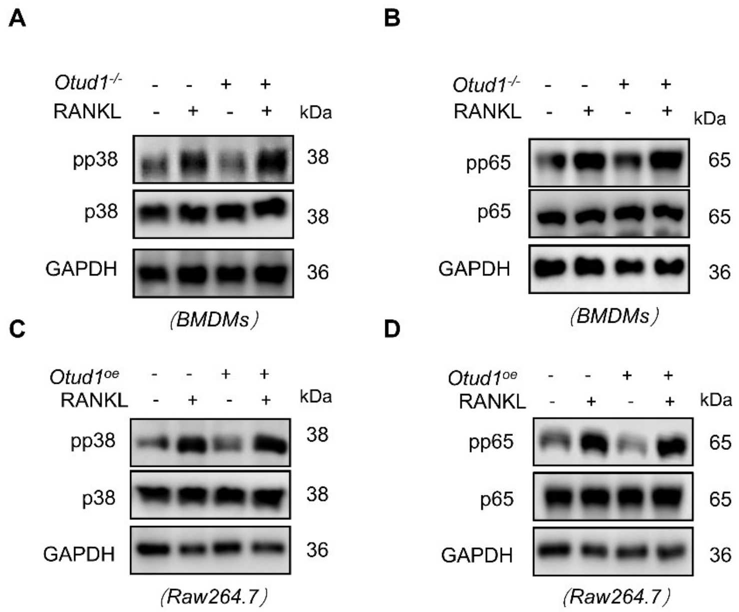

Figure S5. OTUD1 deficiency did not affect the levels of phospho-p65 and p38 during osteoclast differentiation.

(A-B) Representative immunoblot of pp38 and pp65 levels in BMDMs from *Otud1*$^{+/+}$ and *Otud1*$^{-/-}$ *mice* upon RANKL stimulation. **(C-D)** Representative immunoblot of pp38 and pp65 levels in OTUD1-overexpressing and control RAW264.7 upon RANKL stimulation.

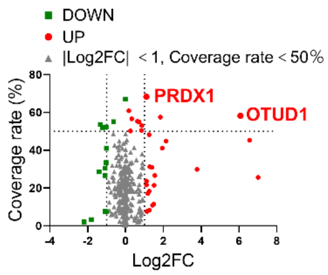

Figure S6. Potential OTUD1-interacting proteins identified through proteomic screening.

Differentially expressed OTUD1-binding proteins analyzed by volcano plots, comparing OTUD1 immunoprecipitation (OTUD1-IP) and IgG-IP groups, with coverage rate on the y-axis and Log2FC of quantified proteins on the x-axis.

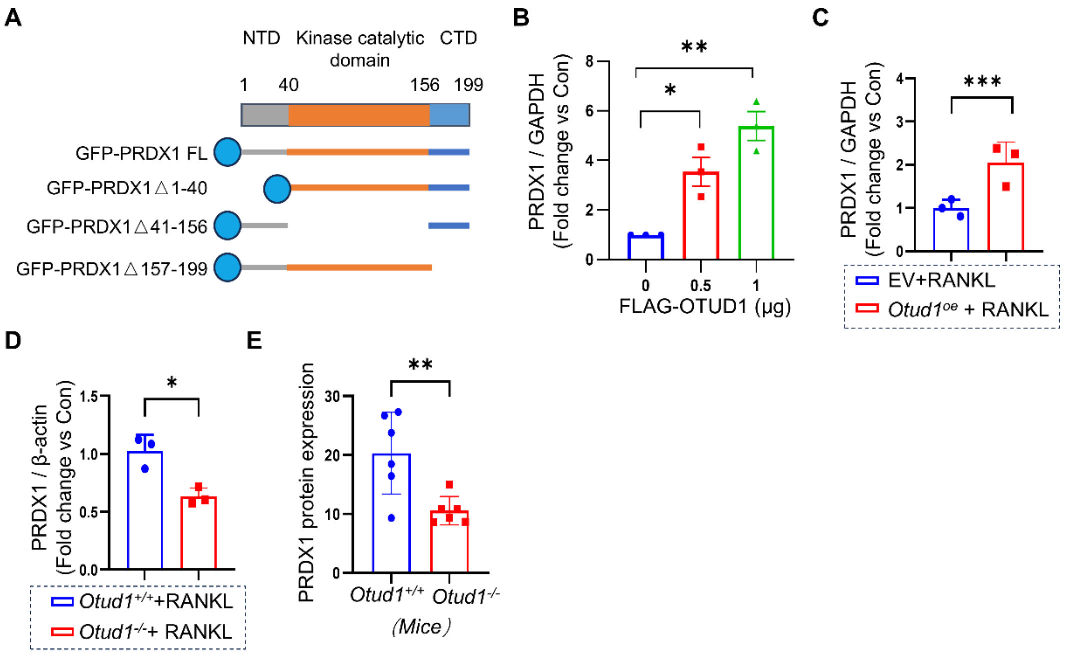

Figure S7. OTUD1 stabilized the protein expression of PRDX1.

(A) Schematic illustration of the PRDX1 domain deletion construct. **(B)** Representative quantification of OTUD1 and PRDX1 in HEK-293T cells transfected with overexpression plasmids of Flag-OTUD1 (*n* = 3). **(C)** Representative quantification of PRDX1 level in RAW 264.7 cells with OTUD1 overexpression (*n* = 3). **(D)** Representative quantification **(G)** of PRDX1 levels in BMDMs from *Otud1*$^{+/+}$ and

Otud1$^{-/-}$ mice (n = 3). **(E)** Quantification of PRDX1 level on trabecular bone surface in distal femur from *Otud1*$^{+/+}$ and *Otud1*$^{-/-}$ mice (n = 6, 100 μm). Data are presented as the mean ± SEM. $*p < 0.05$, $**p < 0.01$, $***p < 0.001$.

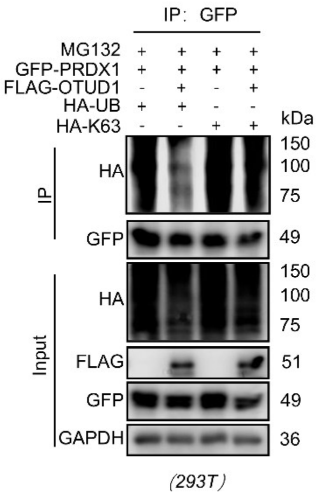

Figure S8. OTUD1 did not affect K63-linked ubiquitination of PRDX1. Immunoprecipitation of PRDX1 in NC or *Otud1*^{oe} 293T cells that co-transfected with overexpression plasmids of GFP-PRDX1, HA-Ub and HA-K63 (K63 only) and then subjected to MG132 (10 μm). Ubiquitinated PRDX1 was detected by immunoblotting via using an GFP-specific antibody to clarify the ubiquitination pattern of PRDX1 regulated by OTUD1.

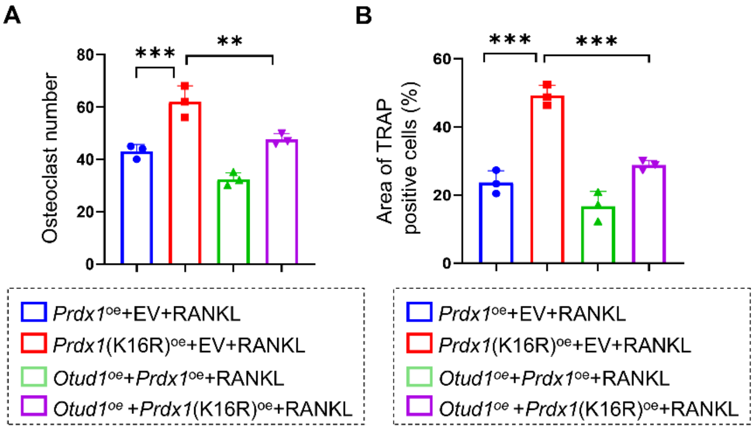

Figure S9. Transfection with the PRDX1 (K16R) mutated plasmid abolished the protective effects of OTUD1 on osteoclast differentiation.

(A) The number of TRAP-positive multinucleated cells upon different treatment. **(B)** Areas of TRAP-positive cells upon different treatment. Data are presented as the mean ± SEM. $^{**}p < 0.01$, $^{***}p < 0.001$.

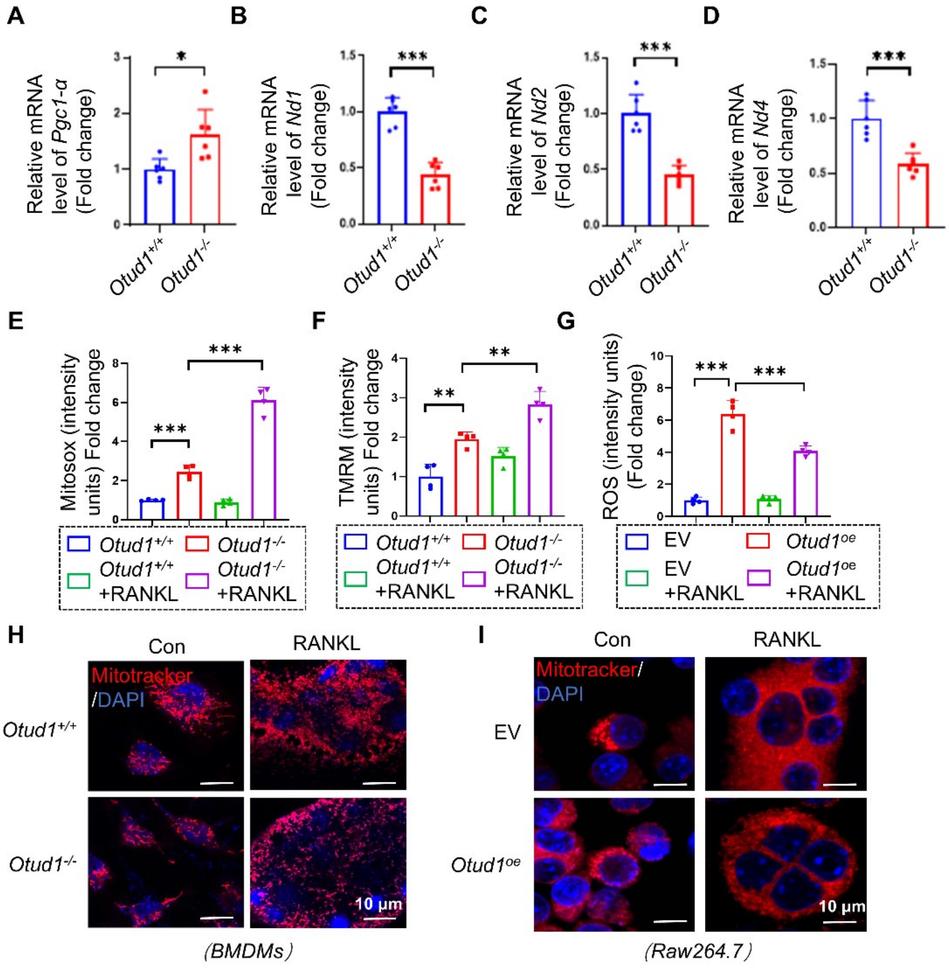

Figure S10. OTUD1 regulated mitochondrial dysfunction in bone loss and osteoclastogenesis.

(A-D) Quantitative RT-PCR analysis of osteogenesis genes including *Pgc1-α* **(A)**, *Nd1* **(B)**, *Nd2* **(C)** and *Nd4* **(D)** mRNA levels in femur tissues from $Otud1^{+/+}$ and $Otud1^{-/-}$ mice (n = 6). **(E, F)** Representative quantitative analysis of Mitosox staining **(E)** and TMRM staining **(F)** upon different treatment (n = 4). **(G)** Representative quantitative analysis of DCFH-DA staining of cells upon different treatment (n = 4). **(H-I)** Representative images of Mitotracker staining of cells upon different treatment (10 μm). Data are presented as the mean ± SEM. $^{*}p< 0.05$, $^{**}p< 0.01$, $^{***}p< 0.001$.

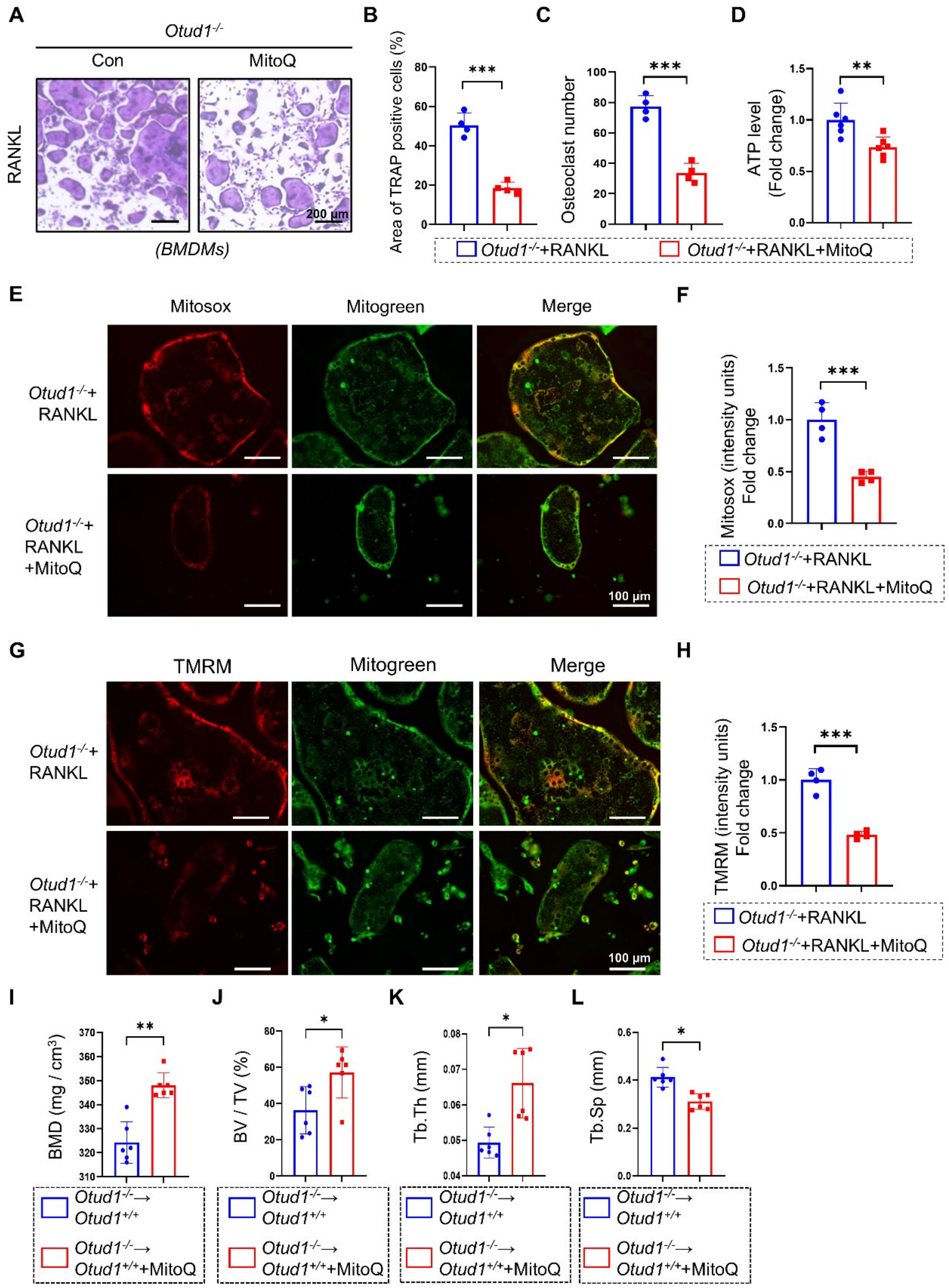

Figure S11. MitoQ reduced osteoclastic differentiation induced by OTUD1 deficiency.

(A-C) Representative image of TRAP staining **(A)** and quantification analysis **(B, C)** of BMDMs from *Otud1*$^{-/-}$ mice after RANKL and MitoQ treatment (*n* = 4, 200 μm). **(D)** The ATP content of BMDMs from *Otud1*$^{-/-}$ mice after RANKL and MitoQ treatment

(n = 6). **(E, F)** Representative Mitosox staining images **(E)** and quantitative analysis **(F)** of BMDMs from *Otud1*$^{-/-}$ mice after RANKL and MitoQ treatment (n = 4, 100 μm). (**G, H**) Representative TMRM staining images **(G)** and quantitative analysis **(H)** of BMDMs from *Otud1*$^{-/-}$ mice after RANKL and MitoQ treatment (n = 4). **(I-L)** Quantification analysis of BMD **(I)**, BV/TV **(J)**, Tb. Th **(K)** and Tb. Sp **(L)** (n = 6, 100 μm). Data are presented as the mean ± SEM. *p< 0.05, **p < 0.01, ***p < 0.001.

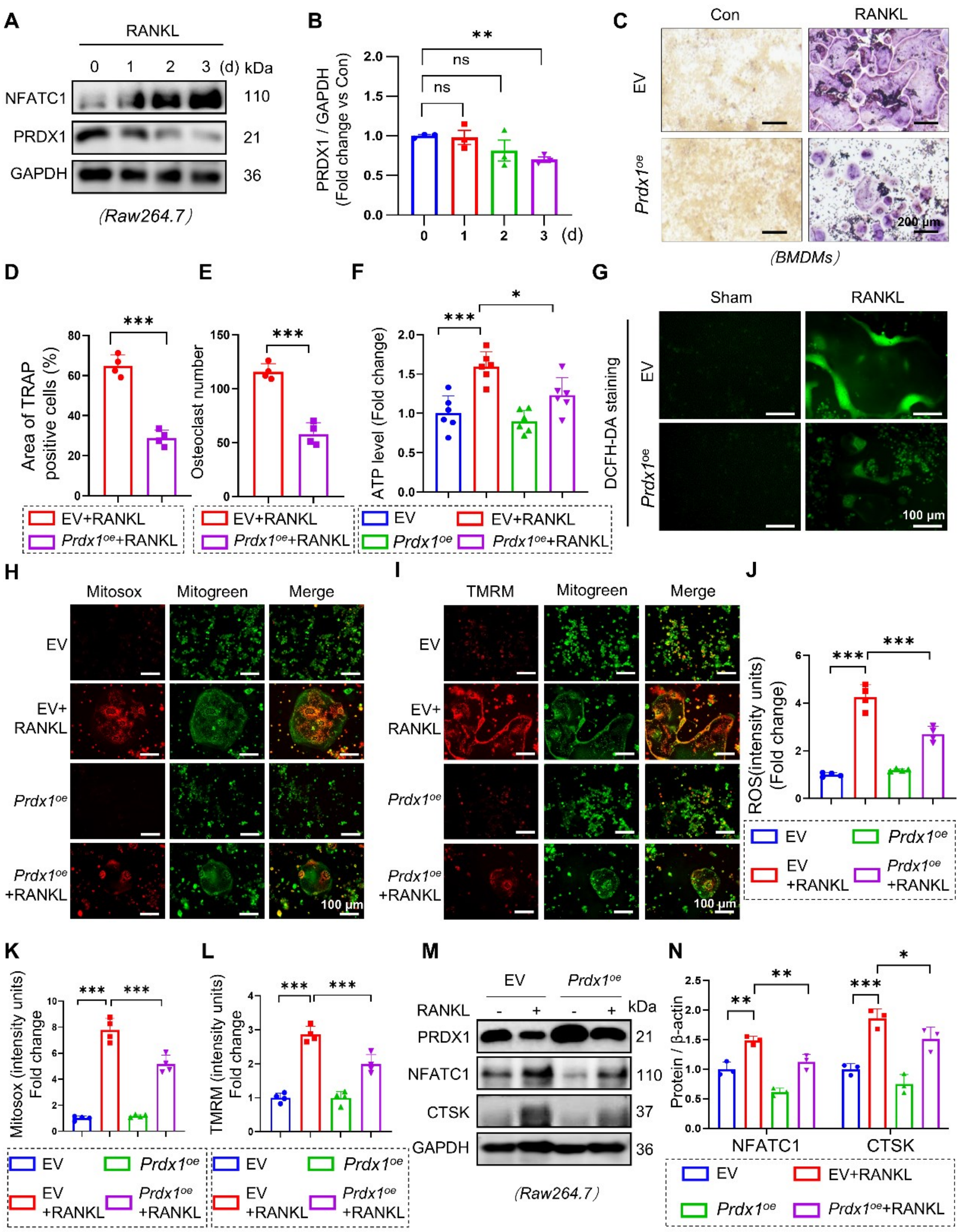

Figure S12. PRDX1 attenuated osteoclastic differentiation and related mitochondrial dysfunction.

(A, B) Representative western blot band **(A)** and quantification **(B)** of PRDX1 levels in RAW264.7 upon different treatments (n = 3). **(C-E)** Representative image of TRAP staining **(C)** and quantification analysis **(D, E)** of PRDX1-overexpressing and control RAW264.7 after RANKL stimulation (n = 4, 200 μm). **(F)** ATP level of RAW264.7

upon different treatments (n = 6). **(G-L)** Representative DCFH-DA staining images **(G)** and quantitative analysis **(J)** of RAW264.7 upon different treatments (n = 4, 100 µm). Representative Mitosox staining images **(H)** and quantitative analysis **(K)** of RAW264.7 upon different treatment (n = 4, 100 µm). Representative TMRM staining images **(I)** and quantitative analysis **(L)** of RAW264.7 upon different treatments (n = 4, 100 µm). **(M, N)** Representative western blot band **(M)** and quantification **(N)** of NFATC1 and CTSK levels in RAW264.7 upon different treatments (n = 3). Data are presented as the mean ± SEM. *$p < 0.05$, **$p < 0.01$, ***$p < 0.001$, ns: no significant.

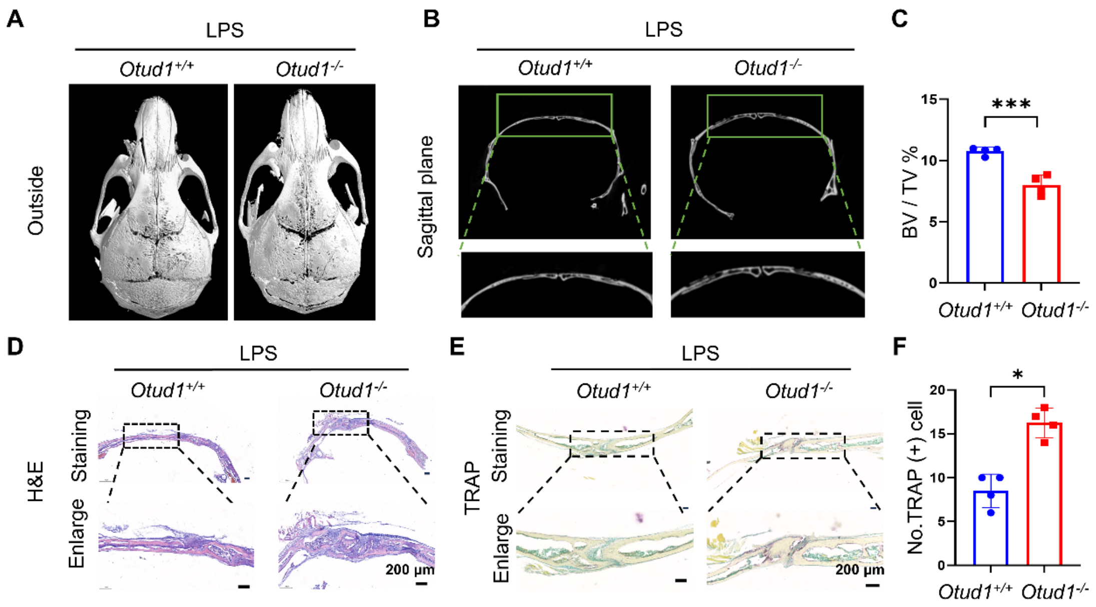

Figure S13. OTUD1 deficiency aggravated LPS-induced cranial bone destruction

(A) Representative whole-mount images of the mice calvarial bone were reconstructed by micro-CT scanner (n = 4). **(B)** Representative cross-section images of the mice calvarial bone were reconstructed by micro-CT scanner (n = 4). **(C)** Quantification of BV/TV in calvarial bone (n = 4). **(D)** Representative images of H&E staining on histological sections of mice calvarial bone, with the boxed area in the first panel magnified below (n = 4, 200 µm). **(E)** Representative images of TRAP staining on histological sections of mice calvarial bone, with the boxed area in the first panel magnified below (n = 4, 200 µm). **(F)** Quantification of TRAP-positive cells in TRAP staining (n = 4). Data are presented as the mean ± SEM. *$p < 0.05$, ***$p < 0.001$.

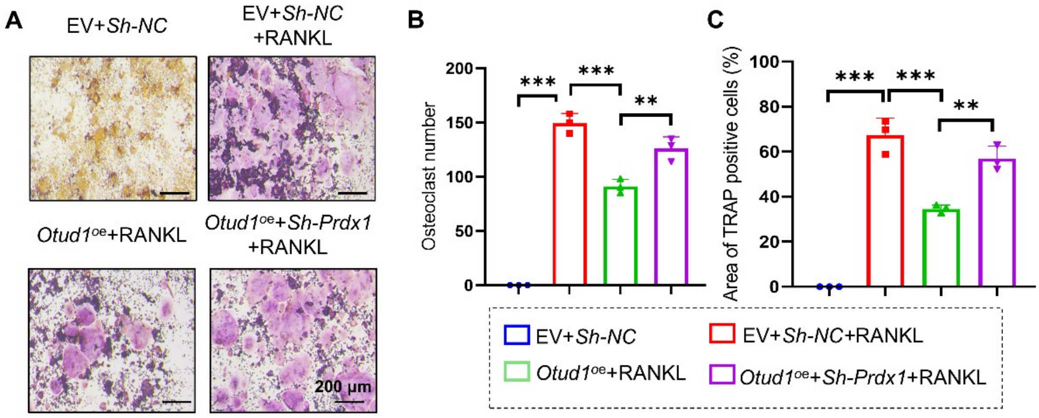

Figure S14. PRDX1 knockdown abolished the protective effect of OTUD1 against osteoclastogenesis.

(A-C) Representative TRAP-stained images of RANKL-induced RAW264.7 cells with OTUD1 overexpression and PRDX1 knockdown **(A)** and quantification analysis of osteoclast number and area of TRAP positive cells **(B, C)** ($n = 4$, 200 μm). $^{**}p < 0.01$, $^{***}p < 0.001$.
